# G-quadruplex DNA reprograms the photochemistry of CX-5461 toward radical-driven anticancer activity

**DOI:** 10.1093/nar/gkag777

**Published:** 2026-08-11

**Authors:** Jakub Trojnar, Maria V Cottini, Marta Dudek, Cyrille Monnereau, Lhoussain Khrouz, Kai-Wei Hsueh, Yen-Chen Liu, Ping-Yen Huang, Jan Jamroskovic, Marco Deiana

**Affiliations:** Faculty of Chemistry, Institute of Advanced Materials, Wrocław University of Science and Technology, Wyb. Wyspiańskiego 27, 50-370 Wrocław, Poland; Department of Microbial Genetics, Institute of Molecular Biology, Slovak Academy of Sciences, Dubravska cesta 21, 845 51 Bratislava, Slovakia; Faculty of Chemistry, Institute of Advanced Materials, Wrocław University of Science and Technology, Wyb. Wyspiańskiego 27, 50-370 Wrocław, Poland; ENS de Lyon, CNRS, Laboratoire de Chimie, UMR 5182, 46 allée d’Italie, F-69342 Lyon, France; ENS de Lyon, CNRS, Laboratoire de Chimie, UMR 5182, 46 allée d’Italie, F-69342 Lyon, France; Clinical Department, Senhwa Biosciences, Inc., 10F, No. 225, Sec. 3, Peihsin Rd., Hsintien Dist., 23143 New Taipei City, Taiwan; Clinical Department, Senhwa Biosciences, Inc., 10F, No. 225, Sec. 3, Peihsin Rd., Hsintien Dist., 23143 New Taipei City, Taiwan; Clinical Department, Senhwa Biosciences, Inc., 10F, No. 225, Sec. 3, Peihsin Rd., Hsintien Dist., 23143 New Taipei City, Taiwan; Department of Microbial Genetics, Institute of Molecular Biology, Slovak Academy of Sciences, Dubravska cesta 21, 845 51 Bratislava, Slovakia; Faculty of Chemistry, Institute of Advanced Materials, Wrocław University of Science and Technology, Wyb. Wyspiańskiego 27, 50-370 Wrocław, Poland

## Abstract

**CX-5461** (pidnarulex) is the most clinically advanced small-molecule ligand associated with G-quadruplex (G4) targeting and shows activity in DNA repair–deficient tumors, yet its clinical use is associated with dose-limiting phototoxicity. Here, we repurpose this intrinsic photoreactivity to investigate **CX-5461** as a G4-associated, light-activated antitumor scaffold. In solution, **CX-5461** produces both Type I and Type II reactive oxygen species (ROS), whereas complexation with G4 DNA markedly decreases detectable singlet oxygen (^1^O_2_) formation and favors radical-associated Type I oxidation. This G4-modulated photochemical shift drives oxidative remodeling and destabilization of G4-containing DNA. In cancer cells, photoactivation enhances cytotoxicity by up to two orders of magnitude relative to the dark state and is accompanied by elevated intracellular ROS, increased 8-oxoG formation, γ-H2AX accumulation, and changes in BG4-detected nuclear G4 structures. *In vivo*, light-activated **CX-5461** suppresses tumor growth and extends survival in immunocompetent syngeneic models, eliciting hallmarks consistent with immunogenic genome stress. Collectively, these findings reposition **CX-5461** as a clinically relevant scaffold for G4-associated photogenomic cancer therapy, while supporting a model in which broader oxidative DNA damage may also contribute to the biological response.

## Graphical Abstract

**Graphical Abstract:**
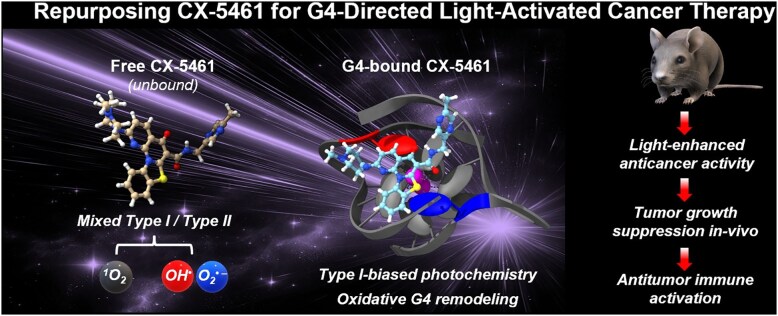

## Introduction

G-quadruplexes (G4s) are guanine-rich, noncanonical nucleic acid secondary structures enriched in gene promoters, telomeres, and replication origins, where they regulate transcription, replication-fork progression, and genome stability [1, 2]. Their prevalence in highly transcribed and replication-stressed genomic regions has positioned G4s as compelling therapeutic targets, particularly in cancers with defective DNA damage response (DDR) pathways [2–6]. Beyond their regulatory roles, the exceptional guanine density of G4 motifs renders them intrinsically susceptible to oxidative modification, marking G4s as privileged substrates for spatially confined oxidative genome damage [7–9]. Despite this convergence of structure, function, and chemistry, clinically actionable strategies that directly exploit G4 reactivity remain scarce [10–15].

**CX-5461** (pidnarulex) is arguably the most clinically advanced small molecule associated with G4 engagement [16]. Initially developed as an inhibitor of RNA polymerase I–driven ribosomal DNA transcription, **CX-5461** was later shown to stabilize G4 DNA, induce replication-associated lesions, and activate ATM/ATR-mediated checkpoint signaling [17–21]. Its high therapeutic activity in homologous recombination–deficient malignancies, including BRCA1/2-mutant breast and ovarian cancers, underscores a synthetic-lethal interaction between G4 stabilization, replication stress, and impaired DDR [17, 22–24]. These properties have motivated clinical evaluation of **CX-5461** in DNA repair–defective solid tumors, positioning it as a rare example of a genome-structure-directed therapeutic agent in advanced clinical development.

Notably, clinical studies also reported dose-limiting phototoxicity upon sun exposure, implicating intrinsic photoreactivity of the **CX-5461** chemotype rather than its canonical synthetic-lethal mechanism [22]. This observation places **CX-5461** at an unusual and largely unexplored crossroad of G4-directed genome stress and photoinduced chemical reactivity. Whether such photoreactivity represents a liability, or a structure-guided therapeutic opportunity, has remained unresolved.

Here we repurpose **CX-5461** as a G4-directed, light-activated antitumor agent. Because **CX-5461** is an established G4-binding ligand, we reasoned that its clinically observed photoreactivity could be exploited to generate oxidative genome stress in proximity to G4-containing regions rather than relying solely on its canonical dark activity. We show that while **CX-5461** generates both Type I and Type II reactive oxygen species (ROS) in solution, complexation with G4 DNA reprograms its photoactivity toward predominantly Type I oxidation. Our data support a model in which photoactivated **CX-5461** induces oxidative alteration of G4-containing DNA, leading to changes in G4 stability, polymerase progression, and cellular markers of genome stress. In cancer cells, photoactivation induces up to a two-orders-of-magnitude enhancement in cytotoxicity, which correlates with elevated intracellular ROS levels, increased 8-oxoG formation, and γ-H2AX accumulation, ultimately leading to pronounced nuclear G4 remodeling. In immunocompetent syngeneic models, light-activated **CX-5461** suppresses tumor growth and extends individual survival while inducing oxidative genome damage associated with immunogenic signaling, including the release of damage-associated molecular patterns (DAMPs) and enhanced CD8⁺ T-cell infiltration. Together, these findings support a G4-associated photogenomic mechanism, while recognizing that broader oxidative DNA damage may also contribute to the cellular and *in vivo* responses.

## Materials and methods

### Spectroscopy

Absorption spectra were recorded using a JASCO V-730 spectrometer (Jasco Inc., USA) equipped with a Jasco Peltier-type temperature controller (CDF-426S/15). All optical measurements were carried out using quartz cuvettes with a standard optical path length of 10 mm.

Fluorescence spectra measurements were performed on a JASCO FP-8550 spectrofluorometer (Jasco Inc., USA), equipped with a Julabo CD-B5 cooling system. All optical measurements were carried out using quartz cuvettes with a standard optical path length of 10 mm.

Singlet oxygen (^1^O_2_) emission spectra were recorded on a Horiba-Jobin-Yvon Fluorolog-3 spectrofluorimeter equipped with a three-slit double-grating excitation and emission monochromator with a dispersion of 2.1 nm/mm (1200 groove/mm) in excitation. Detection was achieved with a liquid N_2_- IR-Symphony II CCD detector, configured with 600/1000/600 g/mm gratings (centered at 1270 nm), Integration time was 10 s.

### ROS generation studies

The ^1^O_2_ quantum yield (*Φ_Δ_*) was determined by a relative method based on near-infrared (NIR) phosphorescence detection of ^1^O_2_. Diluted solutions of **CX-5461** and the reference compound phenalenone (*Φ_Δ_*_,ref_ = 0.95 in CHCl_3_) were prepared in spectroscopic-grade chloroform with optical densities below 0.1 at the excitation wavelength (370 nm) and measured under gentle stirring. The ^1^O_2_ phosphorescence emission was recorded in the 1220–1320 nm range. The detected signal intensity was corrected for dark signal and lamp power fluctuations (T_1C_/R_1C_ correction). *Φ_Δx_* was calculated according to Eq. S1:


(1) \begin{eqnarray*}
{{\phi}_{{\mathrm{\Delta }}x}} = \frac{{{\mathrm{\ }}S_{\mathrm{\Delta }}^x}}{{{\mathrm{\ }}S_{\mathrm{\Delta }}^{\mathrm{ ref}}}}{{\phi}_{{\mathrm{\Delta }}\mathrm{ ref}}},
\end{eqnarray*}

where $S_{\mathrm{\Delta }}^x$and $S_{\mathrm{\Delta }}^{\mathrm{ ref}}$correspond to the slopes obtained from linear fits of the corrected ^1^O_2_ phosphorescence intensity versus absorbance at the excitation wavelength for **CX-5461** and the reference compound, respectively. Measurements were performed at three different concentrations for both sample and reference (Supplementary Fig. S7).

9,10-anthracenediyl-bis(methylene)dimalonic acid (ABDA; MedChemExpress) was used as a ^1^O_2_ probe at a final concentration of 30 μM. Samples were prepared in 50 mM Tris-phosphate buffer (pH = 6.8) with 100 mM KCl and contained 15 μM **CX-5461**, either alone or with the addition of *c-MYC, KIT*, or *VEGF* at final concentrations of 15 μM. Each sample was irradiated with a Hamamatsu Photonics L9588-04 light source at 10% power using a 365 ± 10 nm bandpass filter. Irradiation times ranged from 0 to 900 s, and after each interval, the ABDA absorption spectrum was recorded. A reference cuvette containing all additives was consistently used to compensate for any background signals. The *Φ_Δ_* of **CX-5461** was determined relative to riboflavin sodium phosphate (FMN) (*Φ_Δ_*_(FMN)_ = 0.51 in water) [25]. The kinetics of ABDA photobleaching exhibited first-order behavior, allowing linear regression analysis. *Φ_Δ_* was calculated by integrating the absorption band from 355 to 375 nm and comparing the bleaching rates and integrated absorbances of **CX-5461** and FMN, as described in Eq. S2:


(2) \begin{eqnarray*}
{{{\mathrm{\Phi }}}_{{\mathrm{\Delta }}\left( {\mathrm{ CX} - 5461} \right)}} = {{{\mathrm{\Phi }}}_{{\mathrm{\Delta }}\left( {\mathrm{ FMN}} \right)}} \times \frac{{{{k}_{\mathrm{ CX} - 5461}}}}{{{{k}_{\mathrm{ FMN}}}}} \times \frac{{\mathop \smallint \nolimits_{355}^{375} {{A}_{\mathrm{ FMN}}}\left( \lambda \right)d\lambda }}{{\mathop \smallint \nolimits_{355}^{375} {{A}_{\mathrm{ CX} - 5461}}\left( \lambda \right)d\lambda }}\\,
\end{eqnarray*}

where *k***_CX-5461_** and *k*_FMN_ denote the ABDA bleaching rate constants measured in the presence of **CX-5461** and FMN, respectively, and the integrals correspond to the absorbance integrated over the specified wavelength range for FMN and **CX-5461**.

Singlet Oxygen Sensor Green (SOSG; Thermo Fisher) was used as a ^1^O_2_ probe at concentration of 5 μM. Samples were prepared in 50 mM Tris-phosphate buffer (pH = 6.8) with 100 mM KCl and contained 15 μM **CX-5461**, either alone or with the addition of *c-MYC* at final concentrations of 15 μM. Each sample was irradiated with a Hamamatsu Photonics L9588-04 light source at 40% power using a 365 ± 10 nm bandpass filter. Irradiation times ranged from 0 to 900 s, and after each interval, SOSG emission (*λ*_exc_ = 488 nm) was measured. To further substantiate **CX-5461**–mediated ^1^O_2_ generation, experiments were also performed in deuterated water (D_2_O). Background signals from all components were subtracted.

Dihydrorhodamine-123 (DHR-123; MedChemExpress) was used as a superoxide (O_2_^•–^) probe at a final concentration of 5 μM. Samples were prepared in 50 mM Tris-phosphate buffer (pH = 6.8) with 100 mM KCl and contained 15 μM **CX-5461**, either alone or with the addition of *c-MYC* or *KIT* at final concentrations of 15 μM. Each sample was irradiated with a Hamamatsu Photonics L9588-04 light source at 40% power using a 365 ± 10 nm bandpass filter. Irradiation times ranged from 0 to 900 s, and after each interval, DHR-123 emission (*λ*_exc_ = 488 nm) was measured. Background signals from all components were subtracted.

Hydroxyphenyl fluorescein (HPF; MedChemExpress) was used as a hydroxyl radical (OH^•^) probe at a final concentration of 5 μM. Because HPF can also respond to other highly reactive oxidants, the observed fluorescence signal should be interpreted cautiously and does not provide unambiguous evidence of OH^•^ formation. Samples were prepared in 50 mM Tris-phosphate buffer (pH = 6.8) with 100 mM KCl and contained 15 μM **CX-5461**, either alone or with the addition of *c-MYC, KIT*, or *VEGF* at final concentrations of 15 μM. Each sample was irradiated with a Hamamatsu Photonics L9588-04 light source at 40% power using a 365 ± 10 nm bandpass filter. Irradiation times ranged from 0 to 900 s, and after each interval, HPF emission (*λ*_exc_ = 490 nm) was measured. Background signals from all components were subtracted.

Electron paramagnetic resonance (EPR) spectroscopy was performed at room temperature (RT) on a Bruker E500 X-band spectrometer (*ν* = 9.44 GHz) equipped with a rectangular resonator and operated at a modulation frequency of 100 kHz. Samples containing 2,2,6,6-tetramethyl-4-piperidine (TEMP, Sigma–Aldrich, 10^−2^ M) in chloroform and 5,5-dimethyl-1-pyrroline N-oxide (DMPO, TCI Chemicals, 10^−2^ M) in dimethyl sulfoxide (DMSO) were prepared with **CX-5461** or phenalenone (with TEMP only), adjusted to an absorbance of 1.0 at 365 nm. The samples were prepared under air and loaded into capillary tubes. Irradiation with a 365 nm LED (Thorlabs) was carried out directly within the EPR cavity during spectral acquisition. Instrumental parameters were as follows: microwave power, 69 mW; modulation amplitude, 1 G. Hyperfine coupling constants (a) and *g*-values were obtained by simulation of the experimental spectra using EasySpin (MATLAB toolbox).

### DNA binding studies

Absorption and emission titration experiments were performed with **CX-5461** (10 and 5 μM for absorption and emission measurements, respectively) in 50 mM Tris–phosphate buffer (pH 6.8) supplemented with 100 mM KCl at 25°C. G4 DNA was added incrementally, and samples were equilibrated for 5 min after each addition prior to measurement.

For Job plot analysis, mixtures of **CX-5461** and folded G4 DNA were prepared at different molar fractions while keeping the total concentration of **CX-5461** plus G4 DNA constant at 10 μM. Job plots were recorded for *c-MYC* and *KIT* G4s in 50 mM Tris–phosphate buffer, pH 6.8, containing 100 mM KCl at 25°C. The data were plotted as a function of the mole fraction of **CX-5461** to estimate the apparent binding stoichiometry.

Cy5-quenching experiments were performed using *c-MYC* G4 labeled with Cy5 at either the 5′ or 3′ terminus. Labeled oligonucleotides were prepared at a final concentration of 10 nM in 50 mM Tris–phosphate buffer, pH 6.8, containing 100 mM KCl at 25°C, and titrated with increasing concentrations of **CX-5461**. Samples were equilibrated for 5 min after each addition prior to measurement. Fluorescence values were normalized to the signal recorded in the absence of **CX-5461**, F/F₀, and plotted as a function of **CX-5461** concentration on a logarithmic scale.

Circular dichroism (CD) spectra were recorded on a Jasco J-1500 spectropolarimeter (Jasco Inc., USA) equipped with a Peltier temperature controller (CDF-426S/15) at 25°C. Spectra represent the average of three accumulations. Prior to measurements, the optical chamber was deoxygenated with dry nitrogen and maintained under a nitrogen atmosphere throughout the experiments. Samples were prepared in 10 mM Tris–phosphate buffer (pH 6.8) containing 100 mM KCl and consisted of 2 μM G4 DNA in the presence of **CX-5461** (0–10 μM). Duplex–G4–duplex (DGD) construct was prepared following a stepwise annealing protocol. Briefly, the G-rich strand and the complementary poly-T tether were first separately diluted in aqueous solution containing 100 mM KCl. The G-rich strand was prepared at 100 µM, whereas the poly-T tether was prepared at 120 µM. Each solution was heated to 95°C for 5 min and then allowed to slowly cool to RT overnight by switching off the heating block.

The G-rich strand and the poly-T tether were mixed in a 1:1 volume ratio, giving final strand concentrations of 50 and 60 µM, respectively, to provide a slight excess of the complementary strand. The mixture was then incubated at 4°C for 24 h to promote formation of the duplex arms while preserving the folded G4 core. Prior to analysis, the preassembled DGD constructs were diluted to the desired working concentration, 1 µM with respect to the G-rich strand, in 10 mM Tris–phosphate buffer (pH 6.8) containing 100 mM KCl.

Irradiation was performed using a Hamamatsu L9588-04 light source operated at 95% power with a 365 ± 10 nm bandpass filter, applying exposure intervals ranging from 0 to 65 min. Appropriate baseline references were subtracted from all CD spectra.

CD melting experiments were performed on a Jasco J-1500 spectropolarimeter (Jasco Inc., USA) equipped with a Peltier temperature controller (CDF-426S/15). CD spectra were recorded over the temperature range 20°C–110°C. CD melting studies were conducted at a fixed *c-MYC* concentration (2 μM) either in the absence or in the presence of **CX-5461** (2, 4, or 10 μM). Samples were prepared in 10 mM Tris–phosphate buffer (pH 6.8) containing 10 mM KCl. Where indicated, samples were preirradiated using a Hamamatsu L9588-04 light source operated at 95% power with a 365 ± 10 nm bandpass filter for 65 min prior to CD measurements.

Melting profiles were monitored using the CD signal at 265 nm. For comparison of thermal transition profiles, each trace was normalized between its initial and final CD values according to:


(3) \begin{eqnarray*}
\mathrm{ C}{{\mathrm{ D}}_{\mathrm{nomr}{\mathrm{\ }}\left( T \right)}} = {\mathrm{\ }}\frac{{\left( {\mathrm{ C}{{\mathrm{ D}}_T} - \mathrm{ C}{{\mathrm{ D}}_{\mathrm{final}}}} \right)}}{{\left( {\mathrm{ C}{{\mathrm{ D}}_{\mathrm{initial}}} - \mathrm{ C}{{\mathrm{ D}}_{\mathrm{final}}}} \right)}}{\mathrm{\ }},
\end{eqnarray*}

where CD_*T*_ is the CD signal at temperature *T*, CD_initial_ is the starting CD signal before the thermal ramp at 20°C, and CD_final_ is the signal at the end of the thermal ramp at 110°C. Thus, CD_norm_ = 1 corresponds to the starting signal of each individual sample, and CD_norm_ = 0 corresponds to the final signal of that same sample. The normalized values were used to compare transition profiles and should not be interpreted as absolute folded fractions, particularly for irradiated samples in which photoactivation already attenuated the CD signal before heating. Transition melting values were estimated by sigmoidal nonlinear fitting.

### ^1^H NMR experiments

^1^H NMR measurements were performed at 298 K using a Bruker 600 MHz spectrometer. Samples were prepared in 10 mM Tris–HCl buffer, pH 6.8, containing 70 mM KCl and 10% D_2_O. For **CX-5461** titration experiments, the *c-MYC* G4-forming oligonucleotide was used at a fixed final concentration of 200 μM and titrated with increasing concentrations of **CX-5461**.

### DNA template preparation

Oligonucleotides were purchased from Sigma–Aldrich and used as received unless otherwise noted. DNA stock solutions were prepared in DNase- and RNase-free ultrapure water and stored at 5°C. Oligonucleotide concentrations were determined by UV–vis spectroscopy using the molar extinction coefficients at 260 nm (*ε*_260_) listed in Supplementary Table S1, calculated with the IDT OligoAnalyzer tool. Prior to use, oligonucleotides in 100 mM KCl were annealed by heating at 95°C for 5 min followed by slow cooling to RT overnight. A complete list of sequences is provided in Supplementary Table S1.

### DNA primer extension assay

A TET-labeled primer (1 µM) was annealed to the *c-MYC* DNA template (2 µM) in 50 mM KCl and 10 mM Tris at RT overnight. The annealed DNA, diluted to 40 nM final concentration when mixed with the reaction solution, was incubated in a buffer containing 10 mM Tris, 8 mM MgCl_2_, and 50 mM KCl. Selected samples were supplemented with the chemical compound **CX-5461** (1 µM), and one of them was exposed to UV irradiation at 365 nm for 30 min using a UV lamp (UV-70W, Ultra-Lum Inc.). The control reaction contained 1% DMSO.

EliZyme™ FAST Taq Polymerase (EZ5005, Elisabeth Pharmacon), at a final concentration of 0.05 U/µl, was added to each sample, and the mixtures were equilibrated at 50°C in a heating block for 1 min. DNA extension was started by the addition of 0.2 mM dNTPs. After 30 min, reactions were stopped by adding an equal volume of stop solution containing 20 mM ethylenediaminetetraacetic acid (EDTA) and 95% formamide (1:1, v/v).

Samples were denatured at 95°C for 5 min and immediately cooled on ice before electrophoresis. Products were then loaded onto a freshly cast denaturing sequencing gel containing 10% polyacrylamide, 8 M urea, 25% formamide, and 1× standard TBE buffer (Tris/borate/EDTA). Electrophoresis was run at 60 W for 1 h 45 min.

The gel was visualized using a Typhoon Scanner FLA 9500 (GE Healthcare) at the Alexa 532 setting. Band intensities were quantified from the raw fluorescence gel images using Image Lab 6.1.0 software (Bio-Rad). After background subtraction, the intensities of the full-length product and the indicated G4-associated arrest products were measured and expressed as percentages of the total fluorescence signal within each lane. The arrest-product signal was calculated as the sum of the indicated truncated extension bands. The term “arrest products” is used operationally to describe truncated primer-extension products accumulated after a fixed extension time and does not imply measurement of polymerase pausing kinetics or rate constants. A complete list of sequences is provided in the Supplementary Data.

### Dot blotting assay

*HIF-1α* and *c-MYC* Pu24T G4-forming oligonucleotides (see Supplementary Table S1) were resuspended in nuclease-free water and diluted to a final concentration of 2 µM in folding buffer containing 10 mM Tris and 100 mM KCl. Samples were incubated overnight at RT to allow G4 folding. **CX-5461** was added to selected samples at a final concentration of 10 or 20 µM, whereas control samples were supplemented with 2% (v/v) DMSO. Where indicated, samples were irradiated at 365 nm for 30 min using a UV-70W lamp (Ultra-Lum Inc., Carson, CA, USA). Following irradiation, **CX-5461** was removed using the Monarch® Polymerase Chain Reaction (PCR) & DNA Cleanup Kit (New England Biolabs) according to the manufacturer’s protocol. Purified oligonucleotides were eluted in folding buffer to obtain a final DNA concentration of 2 µM.

Dot blot experiments were performed as previously described, with minor modifications [10]. Briefly, samples were loaded onto an Amersham Hybond-N+ nylon membrane (Cytiva) using a Bio-Dot microfiltration apparatus (Bio-Rad). After blocking with 5% (w/v) milk in Tris-buffered saline (TBS) containing 10 mM Tris, pH 7.4, and 100 mM KCl, the membrane was incubated sequentially with BG4 antibody (0.1 mg/ml) [26], mouse anti-FLAG antibody (1:5000; Novus Biologicals, #NBP1-97410), and HRP-conjugated anti-mouse IgG antibody (1:5000; R&D Systems, #HAF007). Antibodies were diluted in 1% (w/v) milk/TBS. Each incubation step was followed by three washes with TBST buffer containing 10 mM Tris, pH 7.4, 100 mM KCl, and 0.1% (v/v) Tween 20. Chemiluminescent detection was performed using Radiance Q chemiluminescent substrate (Azure Biosystems, #AC2101), and signals were acquired using an Azure c280 imaging system.

### Cellular cytotoxicity assay

CT26, B16F10, and A375 cells were seeded in 96-well plates and allowed to adhere for 24 h at 37°C in a humidified atmosphere with 5% CO_2_. Seeding densities were 5.0 × 10^3^ cells per well (CT26), 1.0 × 10^3^ cells per well (B16F10), and 5.0 × 10^2^ cells per well (A375). **CX-5461** was prepared as a 10 mM stock solution in NaH_2_PO_4_ solution and serially diluted in complete culture medium to obtain a nine-point concentration series starting at 50 μM using fivefold dilutions. Cells were pretreated with **CX-5461** at the indicated concentrations for 1 h (triplicate wells) prior to 365 nm irradiation (100 mJ cm^−2^). A parallel set of plates remained non-irradiated and served as dark controls. Following irradiation, the treatment medium was replaced with fresh complete medium lacking **CX-5461**, and cells were returned to the incubator. CT26 cells were incubated for a total of 72 h, whereas B16F10 and A375 cells were incubated for 144 h. Cell viability was assessed using the CellTiter-Glo (CTG) assay according to the manufacturer’s instructions. Luminescence was recorded using a microplate reader, and IC_50_ values were determined from dose–response curves.

### Cellular ROS levels analysis

Cells were seeded in 96-well plates at densities of 2.0 × 10^4^ cells per well (A375) or (B16F10) and allowed to adhere for 24 h at 37°C in a humidified incubator with 5% CO_2_. Cells were treated with **CX-5461** at the indicated concentrations (0 μM [vehicle], 2.5, 5, 10, 20, and 40 μM) for 2 h, with each condition performed in triplicate. Cells were not washed with phosphate buffered saline (PBS) before irradiation; instead, photoactivation was performed directly in complete culture medium to preserve the bicarbonate/CO_2_-buffered cellular environment. Cells were then exposed to 365 nm irradiation (100 mJ cm^−2^) or maintained under dark conditions as a control. Intracellular ROS levels were measured immediately after irradiation using the DCFDA/H_2_DCFDA assay kit (Abcam, Cat. No. ab113851) according to the manufacturer’s instructions. Fluorescence intensity was recorded using a SpectraMax iD5 microplate reader (*λ*_exc_ = 485 nm, *λ*_em_ = 535 nm). Additional cellular ROS-probe experiments were performed using DHR-123 (MedChemExpress, HY-101894) and HPF (MedChemExpress, HY-111330) to further assess radical-associated oxidative responses after **CX-5461** photoactivation. For DHR-123 experiments, A375 cells were treated with **CX-5461** at 0 μM [vehicle], 3, 10, 30, and 100 μM for 2 h before irradiation or dark incubation. B16F10 cells were treated with **CX-5461** at 0 μM [vehicle], 2.5, 5, 10, 20, and 40 μM for 1 h before irradiation or dark incubation. For HPF experiments, B16F10 cells were treated with **CX-5461** at 0 μM [vehicle], 2.5, 5, 10, 20, and 40 μM for 1 h before irradiation or dark incubation. Fluorescence was recorded using *λ*_exc_ = 515 nm and *λ*_em_ = 536 nm for DHR-123. HPF fluorescence was recorded using *λ*_exc_ = 490 nm and *λ*_em_ = 515 nm. All measurements were performed in triplicate.

### Determine the DAMPs level

CT26 cells were seeded in 48-well plates at a density of 1.0 × 10^5^ cells per well and treated with **CX-5461** for 1 h. Following treatment, cells were exposed to 365 nm irradiation (100 mJ cm^−2^) or maintained under dark conditions as a control. Oxaliplatin (100 μM) was used as a positive control for the DAMPs release. After 48 h of incubation, cell-free supernatants were collected for HMGB1 quantification using an ELISA kit (Chondrex, Cat. No. 6010), and cells were harvested for calreticulin (Abcam, Cat. No. ab209577) analysis by flow cytometry. All measurements were performed in triplicate.

### *In vitro* DC activation

Bone marrow cells were isolated from the femurs and tibias of BALB/c mice. Cells were suspended in RPMI 1640 medium supplemented with 10% FBS, 20 ng/ml GM-CSF, and 10 ng/ml IL-4 at a density of 5 × 10^5^ cells/ml and cultured for 6 days to generate immature bone marrow–derived dendritic cells (iBMDCs). iBMDCs were co-cultured with CT26 cells that had been treated with **CX-5461** alone or **CX-5461** in combination with 365 nm irradiation (100 mJ cm^−2^) for 24 h. Lipopolysaccharide (LPS, 1 µg/ml) was used as a positive control for dendritic cell activation. Following co-culture, BMDCs were stained with LIVE/DEAD™ Fixable violet dead cell stain kit (BV421; Invitrogen, Cat. No. L34964), fluorophore-conjugated antibodies against CD45 (FITC; BioLegend, Cat. No. 103108), CD11c (APC; BioLegend, Cat. No. 117310), CD40 (PE-Cy7; BioLegend, Cat. No. 124622), and analyzed by flow cytometry.

### Immunofluorescence staining

A375 and B16F10 cells were maintained in L-15 medium supplemented with 10% FBS and incubated at 37°C in a humidified atmosphere with 5% CO_2_. Cells were seeded onto coverslips and allowed to adhere for 24 h prior to treatment. Cells were then treated with **CX-5461** (0, 0.03, 0.3, or 1 μM for A375 cells; 0, 1, or 3 μM for B16F10 cells) with or without 365 nm irradiation (100 mJ cm^−2^) for 24 h. For γ-H2AX and BG4 immunofluorescence staining, cells were fixed with 4% paraformaldehyde for 20 min at RT, permeabilized with 0.1% Triton X-100 in PBS, and blocked with 4% bovine serum albumin (BSA) in PBS for 1 h at 37°C. Cells were incubated with anti-γ-H2AX antibody (Cell Signaling Technology, Cat. No. 9718, 1:200) or BG4 antibody (Absolute Antibody, Cat. No. Ab00174-30.126, 1:300) diluted in blocking solution for 2 h at 37°C. For BG4 staining, cells were subsequently incubated with rabbit anti-DYKDDDDK (FLAG) antibody (Cell Signaling Technology, Cat. No. 14 793, 1:600) for 1 h at 37°C. Cells were washed three times with PBS (5 min each) between incubation steps. Secondary labeling was performed using goat anti-rabbit Alexa Fluor Plus 488 (Thermo Fisher Scientific, Cat. No. A32731, 1:1000). For 8-oxoG staining, cells were fixed with ice-cold methanol/acetone (1:1, pre-cooled at −20°C) for 10 min at 4°C and air-dried. Cells were rehydrated in PBS (three washes, 5 min each), followed by DNA denaturation with 1.5 N HCl for 20 min at RT. Cells were then washed with PBS and neutralized with 50 mM Tris–HCl (pH 8.8) for 5 min. After blocking with 4% BSA in PBS (1 h, 37°C), cells were incubated with mouse anti-8-oxoG antibody (Sigma–Aldrich, Cat. No. MAB3560, 1:200), followed by goat anti-mouse Alexa Fluor Plus 555 (Thermo Fisher Scientific, Cat. No. A32727, 1:1000). Nuclei were counterstained with DAPI for 30 min at RT. Fluorescence images were acquired using a Leica SP8 confocal microscope. The foci analysis was processed using ImageJ software.

### Murine tumor models

All animal experiments were conducted in accordance with relevant institutional guidelines and were approved by the respective Institutional Animal Care and Use Committees (IACUCs). The CT26 syngeneic model experiments performed at WuXi AppTec Co., Ltd. were approved under Animal Use Protocol Nos. ON01-GX001-2023v2.1 and ON01-GX001-2025v1.0. The B16F10 syngeneic model experiments performed at the Industrial Technology Research Institute were approved under protocol No. ITRI-IACUC-2025-028. 

#### CT26 model

Female BALB/c mice (6–8 weeks old) were obtained from Shanghai Lingchang Biotechnology Co., Ltd. A colorectal cancer model was established by subcutaneous injection of CT26 cells (3 × 10^5^ cells) into the right flank. Tumor dimensions were measured using digital calipers, and tumor volumes were calculated according to the formula:


\begin{eqnarray*}
{\mathrm{\mathit{ V} = }}\left( {{{{\mathrm{\mathit{ W}}}}^{\mathrm{2}}}{\mathrm{ \times \mathit{ L}}}} \right){\mathrm{/2,}}
\end{eqnarray*}

where *W* represents the short diameter and *L* the long diameter.

When the mean tumor volume reached ~100 mm^3^, mice were randomized into treatment groups (*N* = 5 per group). **CX-5461** was administered intravenously at 25 mg kg^−1^ once weekly along with 365 nm irradiation delivered at 5 J cm^−2^ weekly.

Control groups received vehicle, **CX-5461** alone, or 365 nm light alone. Tumor growth and body weight were monitored three times per week. At the experimental endpoint, mice were sacrificed, and tumors and blood samples were collected for further analyses.

#### CT26 tumor rechallenge experiment

For the tumor rechallenge experiment, one mouse from an independent CT26 cohort treated weekly with **CX-5461** (25 mg kg^−1^, i.v.) plus localized 365 nm irradiation at the tumor site (5 J cm^−2^) achieved complete tumor regression and was selected for rechallenge. This original **CX-5461** + light cohort included five mice, of which one complete responder was available for rechallenge. After complete tumor regression was confirmed, this mouse was rechallenged by subcutaneous injection of CT26 cells (3 × 10^5^ cells) into the contralateral left flank without further **CX-5461** administration or irradiation. Age-matched naïve BALB/c mice were injected subcutaneously with the same number of CT26 cells in parallel and used as controls (*N* = 3). Tumor growth and body weight were monitored three times per week. Tumor volume was calculated as described earlier.

#### B16F10 model

Female C57BL/6 mice (6–8 weeks old) were obtained from the National Center for Biomodels, NIAR, Taiwan. A melanoma model was established by subcutaneous injection of B16F10 cells (3 × 10^5^ cells) into the right flank. Tumor dimensions were measured using digital calipers, and tumor volumes were calculated according to the formula reported above.

When the mean tumor volume reached ~100 mm^3^, mice were randomized into treatment groups (*N* ≥ 6 per group). **CX-5461** was administered intravenously at 25 or 50 mg kg^−1^ once weekly. Mice receiving photoactivation were exposed to a single irradiation dose (365 nm, 5 J cm^−2^) delivered directly to the tumor site on day 1. Control groups received vehicle, **CX-5461** alone, or UVA alone.

Tumor growth and body weight were monitored twice per week, and survival was recorded.

### Immunohistochemistry

CT26 tumors were fixed in formalin, embedded in paraffin, and sectioned. For detection of CD8, deparaffinized 4 µm-thick tissue sections were submitted to heat-induced antigen (Leica, Cat. No. AR9640) retrieval for 20 min at 100°C. Following this, the sections were incubated with an anti-CD8 antibody (Cell Signaling Technology, Cat. No. 98941, 1:200) for 30 min at RT. The signal was developed using 3,3′-diaminobenzidine (Leica, Cat. No. DS9800).

### RNA isolation and real-time qPCR

Total RNA was extracted from CT26 tumors on Day 18 using the RNeasy Mini Kit (Qiagen, Cat. No. 74104) and reverse transcribed into complementary DNA (cDNA) using the HighCapacity cDNA Reverse Transcription Kit (ABI, Cat. No. 4368814). Relative quantification of RNA levels was performed using TaqMan® Gene Expression Master Mix (ABI, Cat. No. 4369016). TaqMan probes used were 18S RNA (Mm03928990_g), IFN-γ (Mm01168134_m1), IFN-β (Mm00439552_s1), and CXCL10 (Mm00445235_m1). CT values were normalized to 18S RNA values. The messenger RNA (mRNA) levels were computed using the ΔΔCT formula and are presented as fold changes compared to a control sample.

### Statistical analysis

Statistical analysis was performed using GraphPad Prism software. Statistical significance was determined based on one-way or two-way analysis of variance (ANOVA). For Kaplan–Meier graphs, statistical significance was determined using the Mantel log-rank test. *P*-values < .05 were considered statistically significant and are represented using the following notations: *P < .05, **P < .01, ***P < .001, and ^****^P < .0001.

## Results and discussion

### G4 binding reprograms the photochemistry of CX-5461 toward radical-driven oxidation

**Figure 1.**
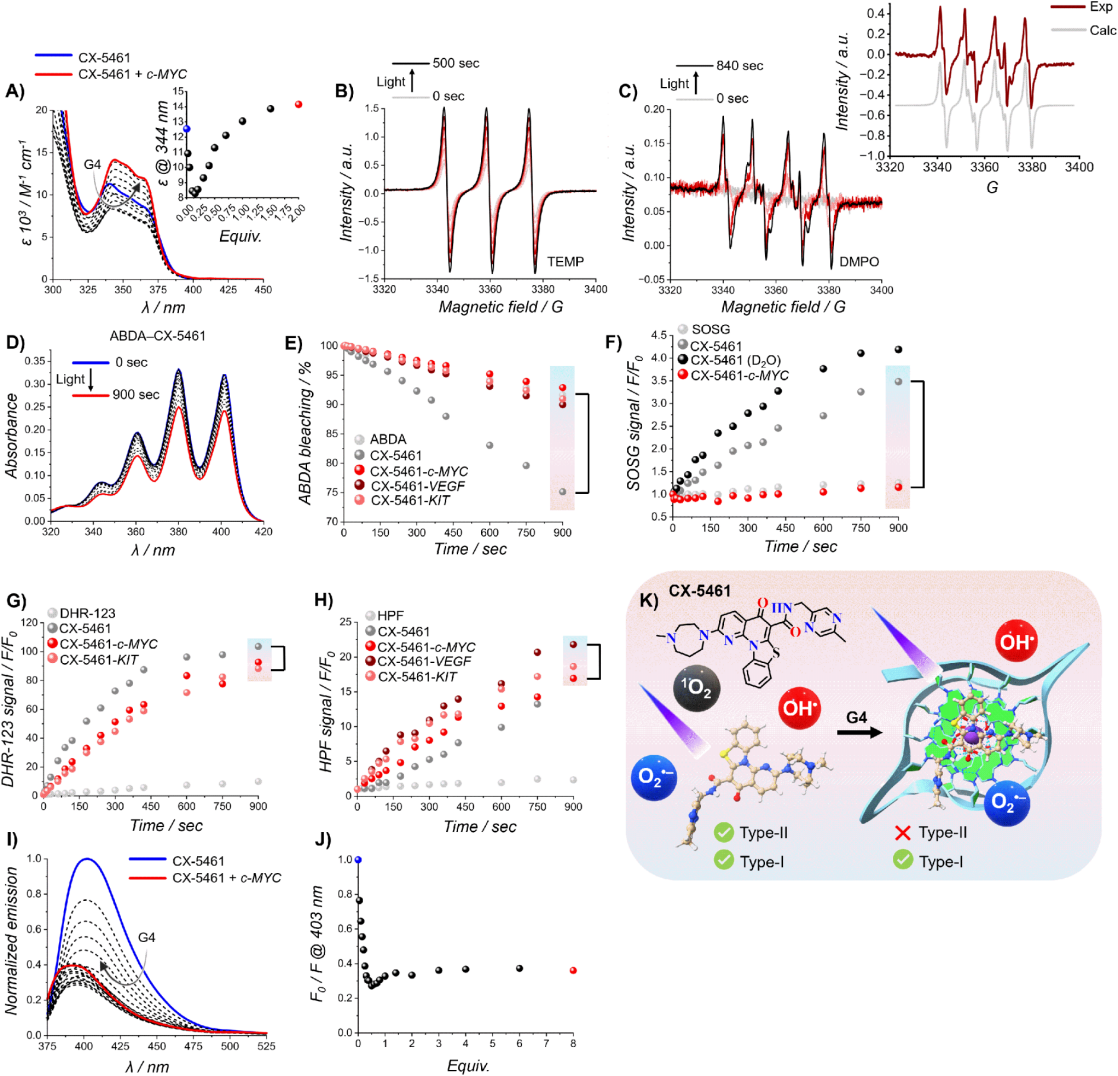
Photophysical and photochemical characterization of **CX-5461**. (**A**) Absorption spectral changes of **CX-5461** (10 μM) upon titration with *c-MYC* G4 DNA (0–20 μM) in Tris buffer (50 mM, pH 6.8, 100 mM KCl). Blue and red traces correspond to 0 and 2.0 equiv. G4 DNA, respectively; dashed lines denote intermediate concentrations. Inset: Changes in molar absorptivity at 344 nm. (**B**) *In situ* EPR detection of ^1^O_2_ using TEMP under 365 nm irradiation in CHCl_3_, showing progressive TEMPO formation. (**C**) *In situ* EPR detection of O_2_^•⁻^ using DMPO under 365 nm irradiation in DMSO; experimental and simulated spectra are shown. (**D, E**) ABDA photobleaching assays monitoring ^1^O_2_ generation by **CX-5461**, free or G4-bound (*c-MYC, KIT, VEGF*), recorded at 380 nm. (**F**) SOSG fluorescence response to ^1^O_2_ upon irradiation of **CX-5461**, free or *c-MYC* G4-bound, including measurements in D_2_O. (**G**) DHR-123 fluorescence detection of O_2_^•⁻^ for free and G4-bound **CX-5461**. (**H**) HPF fluorescence response to highly reactive oxidizing species for free and G4-bound **CX-5461**. All ROS sensor assays were performed in Tris buffer (50 mM, pH 6.8, 100 mM KCl) under 365 nm irradiation using ABDA (30 μM), SOSG (5 μM), DHR-123 (5 μM), HPF (5 μM), **CX-5461** (15 μM), and G4 DNA (15 μM) at 25°C. (**I**) Emission spectral changes of **CX-5461** (5 μM) upon titration with *c-MYC* G4 DNA (0–40 μM) in Tris buffer (50 mM, pH 6.8, 100 mM KCl, *λ*_exc_ = 365 nm). Blue and red traces correspond to 0 and 8.0 equiv. G4 DNA, respectively; dashed lines denote intermediate concentrations. (**J**) Changes in emission intensity at 403 nm. (**K**) Proposed model of **CX-5461** photoactivation, highlighting a transition from mixed Type I/Type II ROS generation in the unbound state to predominantly Type I reactivity upon G4 binding.

**CX-5461** displays a broad near-UV absorption band (*λ*_max_ ≈ 340 nm) with a tail extending to ∼380 nm and an emission maximum centered at ∼405 nm. Notably, the emission intensity is enhanced in aqueous media relative to DMSO or methanol (MeOH), consistent with environment-dependent excited-state behavior (Supplementary Fig. S1). Given its favorable near-UV absorption profile, we next examined the interaction of **CX-5461** with G4 DNA by UV–vis titrations using promoter-derived G4 sequences from *c-MYC, KIT*, and *VEGF* (Supplementary Table S1). The spectral changes evolved over the low-to-equimolar G4 concentration range and reached apparent saturation near one equivalent of G4 DNA, indicating efficient **CX-5461** association under the conditions used here (Fig. 1A and Supplementary Figs S2 and  S3). However, the biphasic optical response suggests that this interaction is unlikely to be described by a single simple binding event. In line with this more complex behavior, Job-plot analyses with *c-MYC* and *KIT* G4s supported an apparent 2:1 ligand stoichiometry, while ^1^H NMR titration of **CX-5461** into *c-MYC* caused broadening of the imino proton resonances without allowing unambiguous assignment of a specific binding site (Supplementary Figs S4 and  S5). Moreover, Cy5-labeled G4 constructs bearing the fluorophore at either the 5′ or 3′ end showed comparable quenching upon **CX-5461** addition, indicating that this readout reports G4 association but does not discriminate between binding at individual outer G-quartets (Supplementary Fig. S6).

Therefore, we interpret the optical titration data as evidence for efficient G4 binding and photophysical modulation of **CX-5461**, rather than as proof of a unique 1:1 complex or a universal preference for one defined terminal tetrad. A plausible binding model involves external G-quartet engagement by the planar aromatic core of **CX-5461**, with additional contributions from loop, groove, or secondary ligand interactions depending on G4 topology and ligand loading. This interpretation is consistent with previous experimental and molecular dynamics studies, which support **CX-5461** binding to G4s and predict multiple G4-associated binding modes, including top, bottom, and side-associated states [17, 27, 28].

Having established efficient **CX-5461** engagement with G4 DNA, we next characterized the intrinsic photochemical output of free **CX-5461** before examining how G4 complexation modifies this behavior.

In chloroform (CHCl_3_), **CX-5461** efficiently generates ^1^O_2_, as confirmed by direct detection of its NIR phosphorescence at 1270 nm (Supplementary Fig. S7). The ^1^O_2_ quantum yield (*Φ_Δ_*) was determined to be 0.22 relative to the reference compound phenalenone. Independent estimates obtained in the same solvent by EPR spectroscopy using TEMP (2,2,6,6-tetramethylpiperidine) as a chemical ^1^O_2_ trap, again with phenalenone as the reference compound, yielded a lower apparent value (*Φ_Δ_* = 0.14). The somewhat lower *Φ_Δ_* obtained from TEMP–EPR measurements likely reflects methodological differences between direct NIR phosphorescence detection and indirect spin-trapping approaches used to monitor ^1^O_2_ formation (Fig. 1B and Supplementary Figs S8–S9) [29, 30].

EPR spin-trapping experiments using DMPO (5,5-dimethyl-1-pyrroline N-oxide) afforded an intense signature associated to the superoxide-derived DMPO–OO(H) adduct upon irradiation of **CX-5461** (*g* = 2.0062, aN = 12.88 G, aH = 10.2 G, aH = 1.45 G), demonstrating that photoexcited **CX-5461** can engage in electron-transfer reactions leading to O_2_^•⁻^ formation and supporting a Type I radical pathway alongside ^1^O_2_ generation (Fig. 1C) [31]. Despite this, no persistent **CX-5461**-centered radicals (N• or C•), indicative of long-lived radical cation intermediates formed following electron transfer, could be detected in the absence of a spin trap (Supplementary Fig. S10).

ROS generation was next evaluated under aqueous buffered conditions compatible with G4 folding. Photooxidation of ABDA, a chemical ^1^O_2_ probe, confirmed ^1^O_2_ production by **CX-5461** (*Φ_Δ_* = 0.13), in excellent agreement with the EPR-derived value (Fig. 1D and E; Supplementary Fig. S11). Independent validation was obtained using SOSG and the characteristic D_2_O enhancement effect arising from the prolonged lifetime of ^1^O_2_ (Fig. 1F and Supplementary Fig. S12).

Strikingly, equimolar complexation with *c-MYC, KIT*, or *VEGF* G4 DNA dramatically suppressed ^1^O_2_ generation (Fig. 1E and F; Supplementary Figs S11 and S12). In contrast, radical-based photochemistry became more prominent. DHR-123, commonly used to monitor oxidizing activity associated with O_2_^•−^-derived pathways, and HPF, widely used to probe OH^•^ radical-associated oxidation, both showed increased fluorescence upon **CX-5461** irradiation, with the HPF response further enhanced upon G4 binding (Fig. 1G and H). However, because HPF can also respond to other highly reactive oxidants, particularly peroxynitrite, and fluorescein-based probes may also be oxidized by secondary radicals such as carbonate radical, the observed signal should be interpreted cautiously and does not provide unambiguous evidence of OH^•^ formation [32, 33]. These observations indicate that G4 complexation markedly decreases the amount of probe-accessible ^1^O_2_ while preserving, and in some cases enhancing, Type I radical-mediated reactivity.

Although the reduced ^1^O_2_ signal could reflect rapid reaction with guanine residues within the G4 structure, thereby preventing its detection by ABDA or SOSG, our previous work and related studies on G4-selective photosensitizers indicate that G4 DNA or RNA can actively modulate photochemical pathways (Type I-to-Type II) [10] and promote radical-driven reactivity [34–36]. Thus, the attenuated ABDA/SOSG response may arise from two non-mutually exclusive effects: rapid local consumption of ^1^O_2_ by solvent-accessible guanine π-faces within the G4 scaffold, and/or a G4-induced change in **CX-5461** excited-state reactivity that reduces the fraction of probe-accessible ^1^O_2_. Consistent with a contribution from the second mechanism, the EPR detection of an O_2_^•⁻^-derived adduct, together with the enhanced DHR-123 and HPF responses, suggests that G4 binding favours electron-transfer processes and downstream radical-associated oxidation in **CX-5461**. Emission studies further supported this view: titration of **CX-5461** with the *c-MYC* G4 sequence resulted in pronounced fluorescence quenching, accompanied at higher G4 concentrations (>0.5 equiv.) by a blue shift of the emission maximum together with a subtle intensity increase. Such structure-dependent changes in the emission profile are consistent with the emergence of an additional excited-state deactivation pathway upon G4 binding, potentially involving electron-transfer interactions between **CX-5461** and the G4 scaffold (Fig. 1I and J). Because Type I photochemistry relies less strictly on molecular oxygen than ^1^O_2_-driven Type II pathways, this G4-biased reactivity profile may be beneficial to photochemical activity under hypoxic conditions typical of solid tumors (Fig. 1K).

### Photoactivation of CX-5461 induces oxidative remodeling of G4 DNA

**Figure 2.**
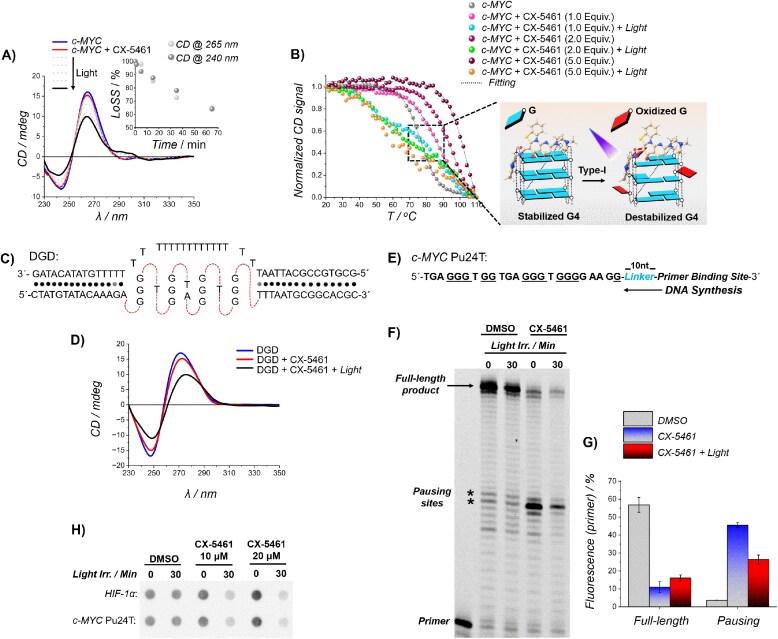
Structural and functional consequences of **CX-5461** photoactivation on the *c-MYC* G4. (**A**) CD spectra of the *c-MYC* G4 recorded in the absence and presence of **CX-5461** under dark conditions or upon 365 nm irradiation. Measurements were performed using *c-MYC* G4 (2 μM) and **CX-5461** (0 or 2 μM) in Tris buffer (10 mM, pH 6.8) containing 100 mM KCl. Inset: loss of secondary structure monitored at 240 and 265 nm. (**B**) CD melting profiles of the *c-MYC* G4 monitored at 265 nm. Measurements were performed using *c-MYC* G4 (2 μM) and **CX-5461** (0, 2, 4, or 10 μM) in Tris buffer (10 mM, pH 6.8) containing 10 mM KCl. Where indicated, samples were preirradiated at 365 nm for 65 min before the thermal ramp. Melting curves are presented as normalized CD intensity at 265 nm. Each trace was normalized between its own initial and final CD values; therefore, the normalized value of 1 reflects the starting CD signal of that individual sample and should not be interpreted as an absolute folded fraction for irradiated samples. (**C**) Sequence of the DGD template used in the assay. Black dots indicate base-paired positions, whereas gray dots indicate mismatched positions. (**D**) CD spectra of the DGD construct recorded in the absence and presence of **CX-5461** under dark conditions or after 65 min of 365 nm irradiation. Measurements were performed using DGD (1 μM) and **CX-5461** (0 or 5 μM) in Tris buffer (10 mM, pH 6.8) containing 100 mM KCl. (**E**) Sequence of the *c-MYC* Pu24T G4 template used in the assay; guanine residues involved in G4 formation are underlined. (**F**) Representative primer extension assay using the *c-MYC* Pu24T G4 template. (**G**) Relative quantification of full-length products and pausing sites derived from panel (F). The DMSO control samples at 0 min irradiation were included in the quantification. Primer extension assay was performed using **CX-5461** (1 μM). Asterisks indicate pausing sites. Irradiation was carried out at 365 nm. Error bars indicate mean ± SD (*N* = 3). (**H**) Dot-blot assay using *c-MYC* Pu24T or *HIF-1α* G4s (2 μM) in the absence or presence of **CX-5461** (10 or 20 μM) under non-irradiated or irradiated conditions. Experiments were performed in 10 mM Tris buffer, pH 7.4, containing 100 mM KCl.

We next examined whether **CX-5461** photoactivation induces structural changes at the DNA level. Under dark conditions, CD spectra of the parallel *c-MYC* and *KIT* G4s showed that **CX-5461** binding largely preserved the characteristic positive band at ∼260 nm and negative band at ∼240 nm, indicating maintenance of the folded G4 architecture (Fig. 2A; Supplementary Figs S13 and  S14). By contrast, irradiation of the preassembled **CX-5461**–G4 complexes caused a pronounced attenuation of the CD signal [10, 37]. This response was **CX-5461**-dependent, as light exposure in the absence of the ligand did not produce comparable spectral changes (Supplementary Fig. S15). We therefore interpret the CD attenuation as photoinduced oxidative remodeling of the ordered G4-containing structure, rather than simple light-induced unfolding of the G4 alone [38–42].

Consistent with this interpretation, the irradiated **CX-5461**–*c-MYC* complex still retained detectable signatures of a parallel G4, including a positive band in the 260–270 nm region and a negative band near 240 nm (Fig. 2A and Supplementary Figs S13 and S14). To determine whether these residual features reflected complete loss of G4 folding, we compared the irradiated spectrum with that of *c-MYC* prepared in the absence of KCl, a condition in which G4 formation is strongly disfavored. The unfolded sample displayed a weak and broad CD profile that was clearly distinct from the irradiated **CX-5461**–G4 spectrum (Figs 2A and Supplementary Figs S13 and S16). In addition, irradiation of the *c-MYC*–**CX-5461** complex generated a weak CD feature around 290 nm that became more pronounced with increasing light exposure. Although its origin cannot be assigned unambiguously, this band is compatible with light-induced remodeling of the G4 ensemble, potentially involving heterogeneous oxidized conformations and altered guanine stacking, in line with previous observations for oxidatively modified G4 sequences [43, 44]. Overall, these data support a model in which **CX-5461** photoactivation partially oxidizes and destabilizes the G4 structure, without fully converting it into an unfolded DNA state.

CD melting experiments further showed that **CX-5461** stabilizes the *c-MYC* G4 in a concentration-dependent manner, with the melting temperature increasing by up to ∼28°C across the tested ligand range (Fig. 2B). This continued stabilization at higher ligand loading is consistent with the complex binding behavior described earlier and argues against a unique, single 1:1 G4 complex [17]. Instead, the data support a model in which **CX-5461** stabilizes the *c-MYC* G4 through one or more G4-associated interactions [27, 28].

To assess whether the same light-triggered structural response occurs in a more genome-like context, we next examined a DGD construct (Fig. 2C) [45, 46]. In this design, the *c-MYC* G4-forming region is flanked by duplex arms, while non-hybridizing positions at the junctions prevent full strand invasion into the G-rich core and help preserve G4 folding. This architecture enables evaluation of **CX-5461** photoactivity in a constrained duplex-embedded G4 environment, where junctional stacking and partial occlusion of terminal tetrads may influence ligand accessibility and binding geometry. The folded DGD construct displayed a CD signature consistent with formation of an ordered G4-containing architecture (Fig. 2D). Upon addition of **CX-5461** under dark conditions, the main DNA CD bands were largely preserved, indicating that ligand binding does not grossly perturb the preformed DGD structure. In contrast, irradiation of the **CX-5461**–DGD complex caused a marked attenuation of the DNA CD signal, consistent with structural alteration of the ordered G4-containing architecture (Fig. 2D). These data indicate that the **CX-5461**-mediated photochemical response is not restricted to short isolated G4-forming oligonucleotides, but is also retained in a duplex-embedded G4 context that better approximates the structural constraints present in genomic DNA.

To directly probe photodamage at G4 sites, we performed a single-nucleotide-resolution DNA primer extension assay (Fig. 2E–G) [4, 10]. In the absence of **CX-5461**, DNA polymerase efficiently bypassed the *c-MYC* G4, and irradiation alone did not substantially affect primer extension, confirming that light by itself does not account for the observed changes in extension products. Addition of **CX-5461** under dark conditions generated a marked arrest site immediately upstream of the first guanine tract, consistent with ligand-induced G4 stabilization [4]. Upon irradiation, this arrest was strongly reduced, indicating that photoactivation of **CX-5461** alters the G4-containing template and compromises the original ligand-stabilized arrest pattern [10]. Because oxidative DNA lesions and strand damage can also influence polymerase progression, the reduced **CX-5461**-dependent arrest after irradiation cannot be assigned solely to G4 unfolding. Instead, together with the CD data, the primer-extension results indicate that **CX-5461** binding enables light-triggered oxidative modification of the G4-containing DNA template, thereby altering the original ligand-stabilized G4 barrier.

To further evaluate whether **CX-5461** photoactivation affects recognition of folded G4 structures, we performed BG4 dot-blot assays using two promoter-derived G4 templates, *c-MYC* Pu24T and *HIF-1α* (Fig. 2H). In both templates, BG4 binding was markedly reduced only after combined **CX-5461** treatment and irradiation, whereas DMSO controls, light-only controls, and **CX-5461**-treated samples kept in the dark retained substantially stronger BG4 signal. These results indicate that **CX-5461** photoactivation decreases BG4 recognition of G4 templates, consistent with oxidative alteration of G4 architecture [10, 47].

### CX-5461 photoactivation induces G4-associated genome stress in cancer cells

**Figure 3.**
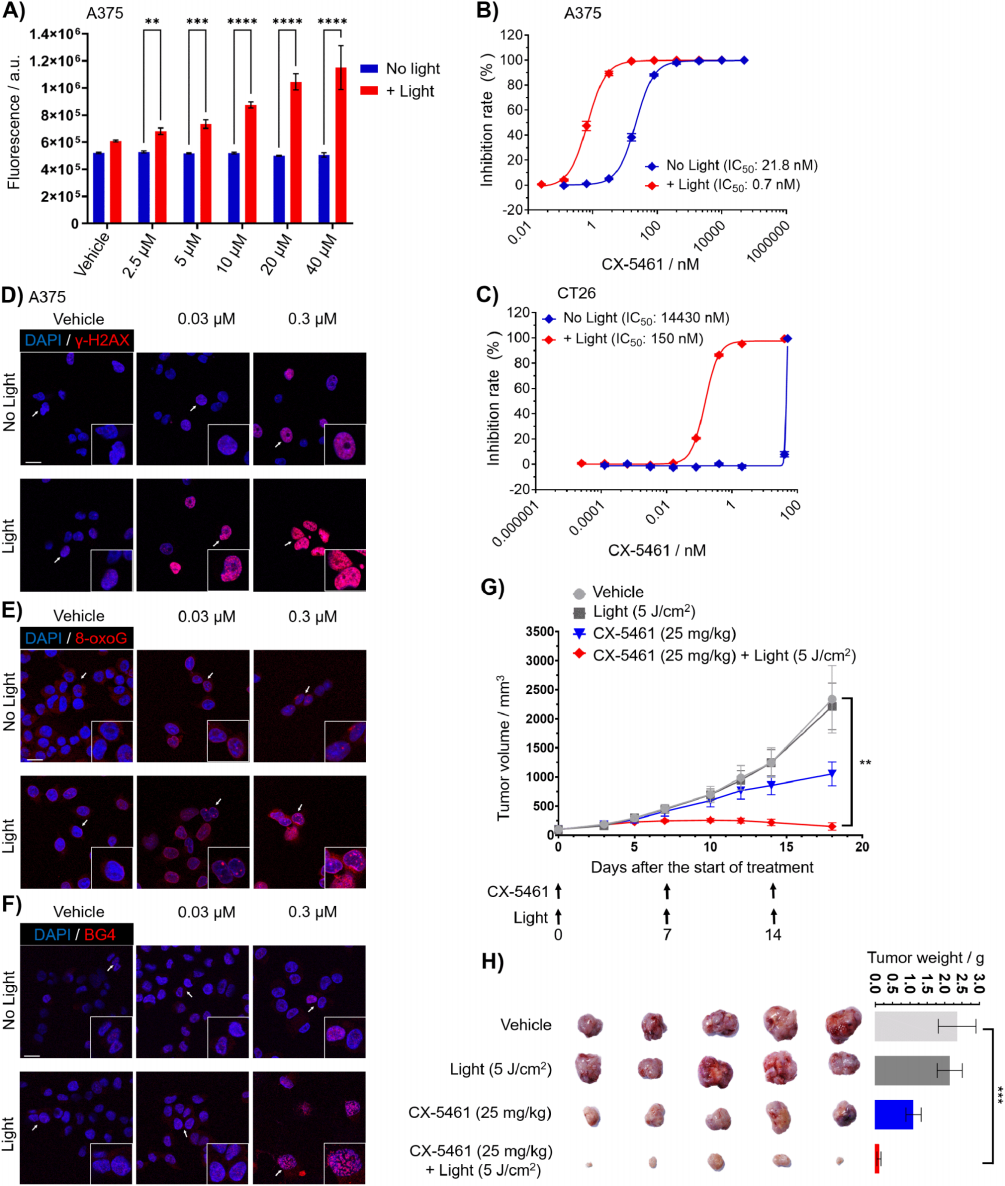
*In vitro* and *in vivo* evaluation of **CX-5461** photoactivation. (**A**) Intracellular ROS generation in A375 cells measured using H_2_DCFDA following **CX-5461** treatment (2 h) under dark and irradiated conditions. Error bars indicate mean ± SD (*N* = 3). Statistical analysis was performed using a two-way ANOVA; *P*-values are indicated (**P < .01; ***P < .001; ^****^P < .0001). (**B, C**) (Photo)cytotoxicity of **CX-5461** in A375 and CT26 cells upon 365 nm irradiation (7 s, 100 mJ cm^−2^). Error bars indicate mean ± SD (*N* = 3). Representative immunofluorescence images showing γ-H2AX foci (**D**), 8-oxoG accumulation (**E**), and nuclear G4 structures detected by BG4 (**F**) in A375 cells treated with **CX-5461** under dark or light conditions (365 nm for 7 s, 100 mJ cm^−2^). Nuclei were counterstained with DAPI. Scale bar: 20 μm. (**G, H**) Antitumor efficacy of **CX-5461** in CT26 tumor-bearing mice under nonirradiated and irradiated conditions. Error bars indicate mean ± SD (*N* = 5). Statistical analysis was performed using a one-way ANOVA; *P*-values are indicated (**P < .01; ***P < .001).

We next evaluated the cellular consequences of **CX-5461** photoactivation in cancer models selected to establish both mechanistic generality and translational relevance. Experiments were conducted in CT26 murine colon carcinoma and B16F10 murine melanoma cells, which align directly with our syngeneic *in vivo* models, as well as in A375 human melanoma cells. Intracellular ROS levels were quantified using the general oxidative stress probe H_2_DCFDA [31]. Cells were irradiated directly in complete culture medium without prior PBS washing to preserve the bicarbonate/CO_2_-buffered cellular environment, which can influence oxidative nucleic-acid chemistry under physiological conditions [48]. **CX-5461** treatment in the dark did not significantly elevate ROS relative to vehicle controls (Fig. 3A and Supplementary Fig. S17). In contrast, irradiation induced a pronounced, concentration-dependent increase in intracellular ROS, consistent with light-triggered oxidative reactivity [12].

Because H_2_DCFDA does not identify individual ROS species, we further examined **CX-5461** photoactivation using additional redox-sensitive probes. SOSG was not used for live-cell measurements because of its limited and variable suitability for intracellular detection, including concerns related to membrane permeability [49, 50]. DHR-123 showed increased fluorescence after irradiation in **CX-5461**-treated A375 and B16F10 cells, consistent with enhanced oxidizing activity associated with O_2_^•⁻^-derived pathways (Supplementary Fig. S18) [51]. HPF fluorescence increased in B16F10 cells following **CX-5461** photoactivation, indicating the formation of highly reactive oxidizing species (Supplementary Fig. S19) [51]. Because HPF is not fully species-selective, this response cannot be attributed specifically to OH^•^. Together with the *in vitro* ROS assays, these cellular probe experiments support the conclusion that **CX-5461** photoactivation enhances intracellular oxidative stress and promotes radical-associated redox chemistry under biologically relevant conditions.

We next evaluated the (photo)cytotoxicity of **CX-5461**. The compound exhibited pronounced light-dependent potency, with up to a 96-fold enhancement in CT26 cells and 31-fold and 12-fold increases in A375 and B16F10 cells, respectively, upon irradiation (Supplementary Table S2, Fig. 3B and C, and Supplementary Fig. S20). Importantly, this light-dependent cytotoxicity was observed under conditions in which **CX-5461** alone produced limited toxicity in the dark. This distinction is relevant because **CX-5461** has established biological activities unrelated to photoactivation, including G4 stabilization-associated replication stress and other genome-interacting mechanisms. By using low **CX-5461** concentrations and short irradiation times, we aimed to minimize nonspecific drug–DNA interactions and reduce the contribution of canonical dark cytotoxic pathways. In this concentration regime, photoinduced toxicity is more likely to reflect activation of **CX-5461** at preferential binding sites, including high-affinity G4 structures, rather than generalized drug toxicity. The comparatively lower enhancement observed in B16F10 melanoma cells likely reflects their high melanin content, which can confer intrinsic photoprotection and increased antioxidant capacity, partially attenuating ROS-mediated phototoxicity.

To directly probe photogenomic damage, we performed immunofluorescence staining in A375 and B16F10 cells using γ-H2AX as a marker of DNA double-strand breaks (DSBs), an anti-8-oxoG antibody to detect oxidative base lesions, and the G4-selective antibody BG4 [52] to visualize nuclear G4 structures (Fig. 3D–F and Supplementary Figs S21–23) [11, 12]. At the concentrations tested, **CX-5461** produced only minimal to moderate effects under dark conditions. In contrast, photoactivation induced a pronounced increase in γ-H2AX *foci*, elevated 8-oxoG accumulation, and a significant enrichment of BG4 nuclear *foci* [12]. These cellular data do not allow assignment of the damage exclusively to individual G4 sites. However, the parallel increase in oxidative DNA lesions, DNA damage signalling, and BG4-detected nuclear G4 structures is consistent with photoinduced oxidative genome stress that includes a G4-associated component.

Although oxidative guanine lesions are commonly associated with local G4 destabilization, the observed BG4 signal amplification is consistent with a damage-driven increase in G4 formation. Oxidative stress and DSB generation promote transient single-stranded DNA regions during replication, transcription, and DNA repair, thereby facilitating refolding of G-rich sequences into G4 structures [12]. In addition, oxidation-induced extrusion of damaged G-tracts, enabled by spare G-runs, may further favor G4 reorganization [53]. Together, these effects rationalize the elevated BG4 *foci* detected following **CX-5461** photoactivation. Thus, the cellular phenotype is best described as G4-associated genome stress rather than simple loss of G4 structures.

### Light-activated CX-5461 induces immunogenic genome stress and suppresses tumor growth in syngeneic models

To assess translational potential, we evaluated antitumor efficacy in immunocompetent syngeneic mouse models of CT26 colon carcinoma and B16F10 melanoma (Fig. 3G and H; Supplementary Figs S24–26). These models were selected to represent the extremes of the observed *in vitro* phototherapeutic window, with CT26 exhibiting the largest enhancement (∼2 orders of magnitude improvement) and B16F10 the most limited light-dependent response (∼1 order of magnitude).

CT26 tumor-bearing mice received weekly intravenous administration of **CX-5461** (25 mg kg^−1^), either alone or combined with localized 365 nm irradiation (5 J cm^−2^) directed to the tumor site (Fig. 3G and H). Control groups included vehicle, non-irradiated **CX-5461**, and light-only treatments.

In the CT26 model, **CX-5461** alone produced only a modest delay in tumor growth, while 365 nm irradiation alone had no measurable effect. In striking contrast, the combination of **CX-5461** and 365 nm irradiation resulted in near-complete suppression of tumor progression over the observation period (14–18 days), consistent with a robust light-dependent therapeutic response *in vivo* (Fig. 3G and H). Body weight remained stable across all groups, indicating the absence of overt systemic toxicity (Supplementary Fig. S24).

In the aggressive B16F10 melanoma model, **CX-5461** treatment under dark conditions produced only limited tumor growth inhibition at both tested doses (25 and 50 mg kg^−1^, i.v.). For photoactivation studies, mice received **CX-5461** (25 or 50 mg kg^−1^) administered weekly and were exposed to a single localized UV dose (5 J cm^−2^) delivered directly to the tumor site (Supplementary Fig. S26). Under these conditions, light activation markedly delayed tumor progression and significantly extended survival relative to the corresponding dark, vehicle, and light-only control groups. Notably, superior tumor control was achieved at the higher **CX-5461** dose (50 mg kg^−1^), which prolonged tumor regression compared with the 25 mg kg^−1^ cohort (Supplementary Fig. S26).

We note that these *in vivo* experiments cannot establish that tumor growth inhibition arises exclusively from oxidative damage at G4 sites. Photoactivated **CX-5461** is expected to induce broader oxidative stress within the irradiated tumor region, and this may contribute to DNA damage, immune activation, and antitumor efficacy. Nevertheless, the use of a clinically advanced G4-binding ligand, together with the *in vitro* evidence that G4 complexation alters **CX-5461** photochemistry and the cellular evidence of oxidative DNA damage accompanied by BG4-detected G4 changes, supports a model in which G4-associated photogenomic stress contributes to the observed therapeutic response.

**Figure 4.**
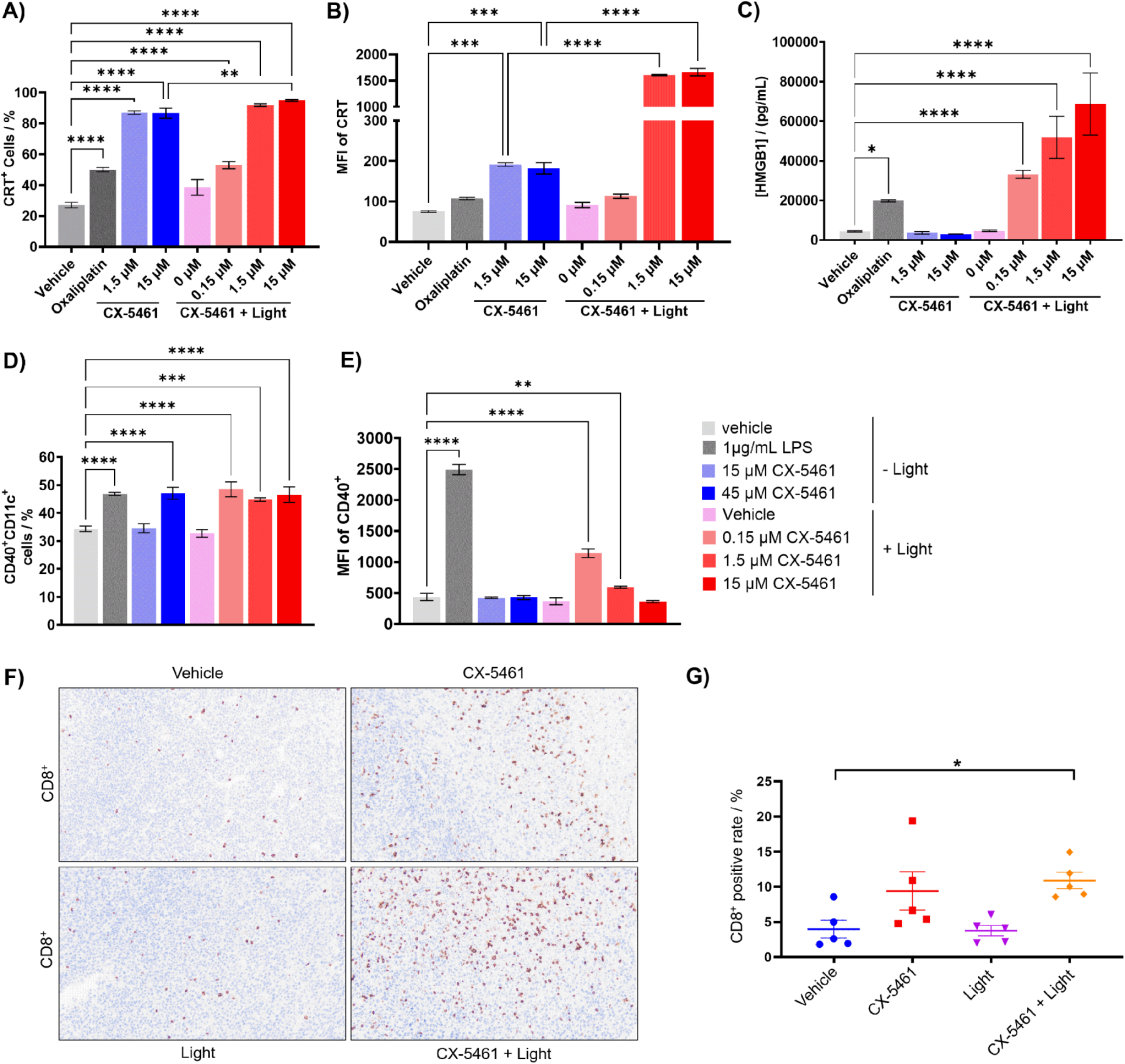
*In vitro* dendritic cell activation and *in vivo* T cell infiltration following **CX-5461** photoactivation. (A–C) ICD markers in CT26 cells treated with **CX-5461** under dark or irradiated conditions (365 nm, 7 s, 100 mJ cm^−2^). (**A**) Percentage of calreticulin-positive (CRT⁺) cells and (**B**) mean fluorescence intensity (MFI) of surface CRT were quantified by flow cytometry. (**C**) Secreted HMGB1 levels were measured by ELISA. Error bars represent mean ± SD (*N* = 5). Statistical analysis was performed using one-way ANOVA (*P < .05; **P < .01; ***P < .001; ^****^P < .0001). (D, E) CT26 cells treated with **CX-5461** under dark or irradiated conditions were co-cultured with iBMDCs for 24 h. (**D**) Percentage of activated dendritic cells (CD11c⁺ CD40⁺) and (**E**) MFI of CD40 expression on dendritic cells were analyzed by flow cytometry. Error bars represent mean ± SD (*N* = 3). Statistical analysis was performed using one-way ANOVA; significance levels are indicated (**P < .01; ***P < .001; ^****^P < .0001). (**F, G**) CT26 tumors were harvested on day 18 and analyzed for CD8⁺ T cell infiltration by immunohistochemistry. Error bars represent mean ± SD (*N* = 5). Statistical analysis was performed using one-way ANOVA (*P < .05).

Given that oxidative genome damage can promote immunogenic tumor stress, and that **CX-5461** has been shown to act as an immunogenic cell death (ICD) compound [54], we next examined DAMPs of ICD induced by **CX-5461** photoactivation. In CT26 cells, **CX-5461** treatment increased surface exposure of calreticulin (CRT), a prototypical “eat-me” signal associated with ICD, with further amplification upon light irradiation (Fig. 4A and B). Notably, **CX-5461** photoactivation elicited CRT externalization that matched or surpassed the response induced by oxaliplatin, included as a canonical ICD-inducing positive control. In parallel, **CX-5461** photoactivation promoted extracellular release of high-mobility group box 1 (HMGB1), which is consistent with oxidative stress signaling (Fig. 4C). Together, these findings indicate that light activation of **CX-5461** induces molecular features characteristic of immunogenic tumor stress [55, 56].

To determine whether **CX-5461**–induced tumor stress translated into an adaptive immune response, we evaluated dendritic cell activation *in vitro* using iBMDCs, with LPS included as a positive control for maturation. CT26 cells treated with **CX-5461** stimulated dendritic cell maturation, which was further enhanced following photoactivation (Fig. 4D and E). Notably, photoactivated **CX-5461** induced the most pronounced dendritic cell activation at lower drug concentrations, consistent with an immunogenic stress window rather than nonspecific cytotoxicity [57].

Finally, quantitative PCR was used to assess activation of the cGAS–STING–type I interferon (IFN) signaling pathway [cyclic GMP–AMP synthase (cGAS)–stimulator of interferon genes (STING)], and immunohistochemical analysis of CD8⁺ cytotoxic T lymphocytes, key effector cells mediating antitumor immunity, was performed in CT26 tumors [58]. These results revealed significant upregulation of IFN and its downstream target gene *CXCL10* (Supplementary Fig. S27). Furthermore, infiltration of CD8⁺ T cells was markedly increased following **CX-5461** photoactivation (Fig. 4F and G). Finally, we asked whether complete tumor regression following **CX-5461** photoactivation was associated with durable antitumor protection. In the CT26 study, one out of five mice in the **CX-5461** + light group achieved complete tumor regression after weekly treatment (Supplementary Fig. S28). This complete responder was subsequently rechallenged with CT26 cells in the contralateral flank, while naïve BALB/c mice were injected in parallel as controls. No tumor regrowth was observed in the rechallenged mouse, whereas naïve mice developed tumors under the same conditions (Supplementary Fig. S28D). Although limited to a single complete responder, this observation is consistent with the induction of protective antitumor immune memory following **CX-5461** photoactivation [59–61].

## Conclusions

In conclusion, we demonstrate that **CX-5461**, currently involved in advanced clinical studies as a G4-targeting and stabilizing small molecule, can be repurposed as a structure-guided, light-activated photogenomic scaffold. G4 binding reprograms **CX-5461** photochemistry toward radical-dominated Type I pathways, decreasing detectable or probe-accessible ^1^O_2_ and enabling oxidative reactivity in G4-containing DNA. Our data indicate that photoactivation converts **CX-5461** from a dark-state G4-stabilizing ligand into a light-responsive oxidizing agent that promotes structural alteration of G4-containing templates and G4-associated genome stress. In cancer cells, this process is accompanied by pronounced phototoxicity, increased intracellular ROS, 8-oxoG formation, γ-H2AX accumulation, and changes in BG4-detected nuclear G4 structures. Importantly, this mechanism translates into robust antitumor efficacy in immunocompetent syngeneic colon and melanoma models, extending survival while inducing hallmarks of immunogenic tumor stress, including DAMPs exposure, IFN elevation, and enhanced CD8^+^ T-cell infiltration in the tumor microenvironment, which demonstrates significant immune antitumor efficacy for tumor inhibition.

We acknowledge that the cellular and *in vivo* effects cannot be assigned exclusively to oxidative damage at individual G4 sites, and that broader oxidative DNA damage likely contributes to the biological response. Nevertheless, the combined photochemical, biophysical, biochemical, cellular, and *in vivo* data support a model in which G4-bound or G4-proximal **CX-5461** contributes to photoinduced oxidative genome stress.

Collectively, these findings establish a general strategy for exploiting genome-structure-encoded reactivity through light-controlled activation of clinically relevant small molecules and highlight opportunities to transform G4-binding chemotypes into photogenomic therapeutics.

## Supplementary Material

gkag777_Supplemental_File

## Data Availability

The data underlying this article are available in the article and in its online supplementary material.
