# Supplementary material for: G-quadruplex DNA reprograms the photochemistry of CX-5461 toward radical-driven anticancer activity
Source: Nucleic Acids Res. 2026 Aug 11;54(15):gkag777. doi: 10.1093/nar/gkag777 (PMC13458403; doi:10.1093/nar/gkag777)
Supplement: Supplementary file 1 [file gkag777_supplemental_file.pdf]

Supporting Information

G-quadruplex DNA reprograms the photochemistry of CX-5461 toward radical-driven anticancer activity

Jakub Trojnar[1], Maria V. Cottini[2], Marta Dudek[1], Cyrille Monnereau[3], Lhoussain Khrouz[3], Kai-Wei Hsueh[4], Yen-Chen Liu[4], Ping-Yen Huang[4], Jan Jamroskovic[2] and Marco Deiana[1,*]

[1] Institute of Advanced Materials, Faculty of Chemistry, Wrocław University of Science and Technology, Wyb. Wyspiańskie-go 27, 50-370 Wrocław, Poland
[2] Department of Microbial Genetics, Institute of Molecular Biology, Slovak Academy of Sciences, Dubravska cesta 21, 845 51 Bratislava, Slovakia
[3] ENS de Lyon, CNRS, Laboratoire de Chimie, UMR 5182, 46 allée d'Italie, F-69342 Lyon, France
[4] Senhwa Biosciences, Inc., 10F, No. 225, Sec. 3, Peihsin Rd., Hsintien Dist., 23143 New Taipei City, Taiwan

* To whom correspondence should be addressed. Email: m.deiana@pwr.edu.pl

Synthesis

CX-5461 was synthesized according to previously published procedures.(1)

Oligonucleotide sequences used for biophysical studies

Table S1. DNA oligonucleotides used in biophysical and dot blot studies.

Name	Sequence *5´- 3´*	Length [bp]	ε_{260} [$M^{-1}cm^{-1}$]	GC [%]	Topology
c-MYC	TGAGGGTGGGTAGGGTGGGTAA	22	228700	59.1	parallel
KIT	CGGGCGGGCGCGAGGGAGGGT	21	204000	85.7	parallel
VEGF	CGGGGCGGGCCTTGGGCGGGGT	22	200400	86.4	parallel
HIF-1α	GCGCGGGGAGGGGAGAGGGGGCGGGAGCGCG	31	309400	87.1	parallel
***c-MYC* Pu24T**	TGAGGGTGGTGAGGGTGGGGAAGG	25	248200	68.0	parallel
G4 strand	CTATGTATACAAAGAGGGTGGGTAGGGTGGGTTT AATGCGGCACGC	46	458900	52.2	parallel
Poly-T	GCGTGCCGCATTAATTTTTTTTTTTTTTTTTTTTTTT GTATACATAG	46	417400	23.9	-
Cy5-c-MYC	[Cy5]TGAGGGTGGGTAGGGTGGGTAA	22	-	59.1	parallel
c-MYC-Cy5	TGAGGGTGGGTAGGGTGGGTAA[Cy5]	22	-	59.1	parallel

Oligonucleotide sequences used for DNA primer extension assay

Primer (5´–to–3´):

TET-TGAAAACATTATTAATGGCGTCGAGCGTCCG

c-MYC Pu24T template (5´–to–3´):

TGA**GGG**T**GG**TGA**GGG**T**GGG**GAAG**G**ATATATATATCGGACGCTCGACGCCATTAATAATGTTTTCA

Cellular (photo)cytotoxicity assay

Table S2. CX-5461 mediated cellular (photo)toxicity.

Cell line	IC_{50} (dark) / nM	IC_{50} (light) / nM	Ratio (dark/light)
CT26	14430	150	96.2
B16F10	947.9	81.4	11.6
A375	21.8	0.7	31.1

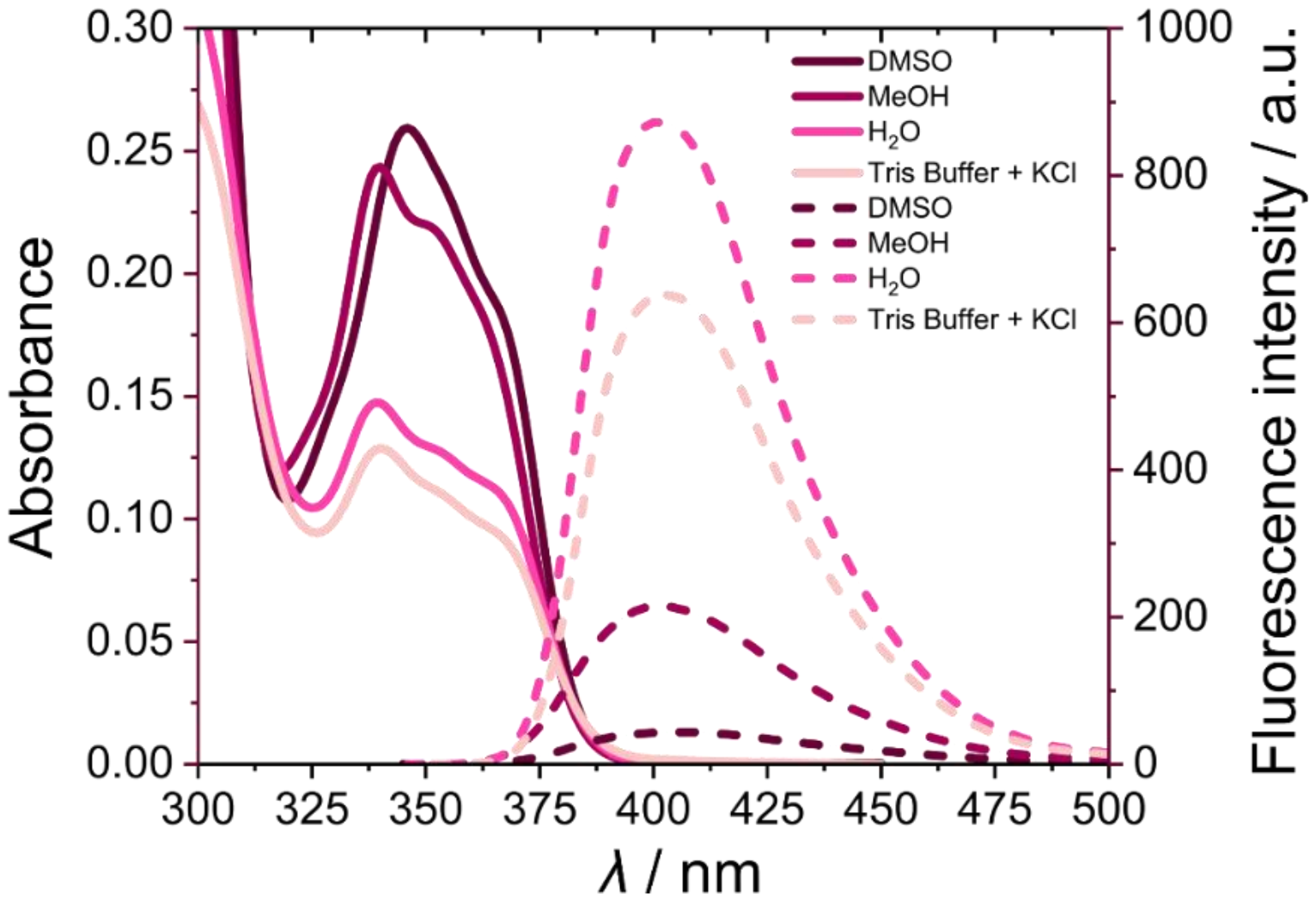

Figure S1. Absorption (solid lines) and fluorescence emission (dashed lines) spectra of **CX-5461** (5 µM) recorded in DMSO, MeOH, H_2O, and aqueous buffer (50 mM Tris, pH 6.8, 100 mM KCl). Emission spectra were obtained upon excitation at λ_{exc} = 340 nm.

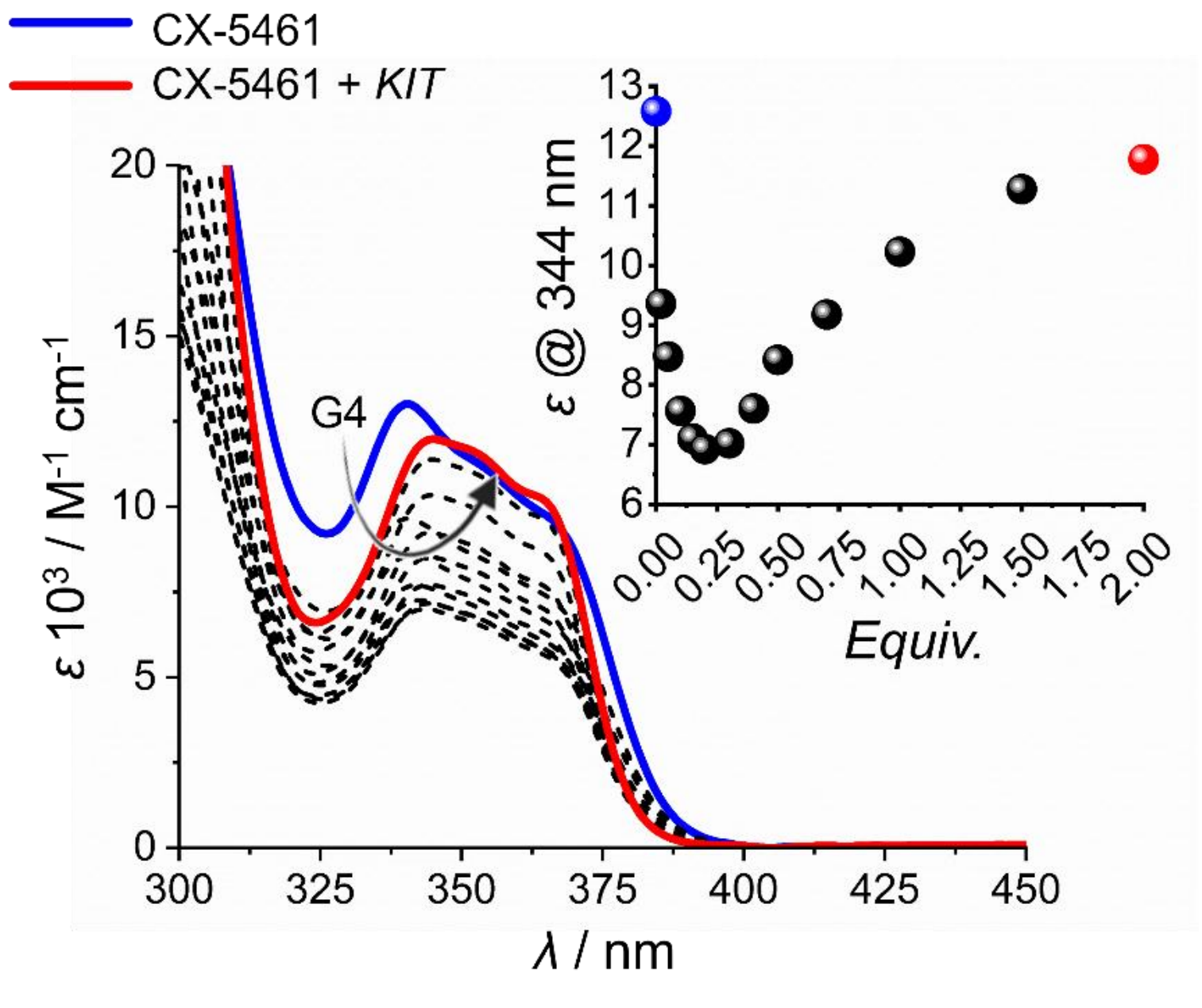

Figure S2. Absorption spectral changes of **CX-5461** (10 µM) upon titration with *KIT* G4 DNA (0–20 µM) in Tris buffer (50 mM, pH 6.8, 100 mM KCl). Blue and red traces correspond to 0 and 2.0 equiv of *KIT* G4 DNA, respectively; dashed lines represent intermediate concentrations. Inset: Changes in molar absorptivity at 344 nm.

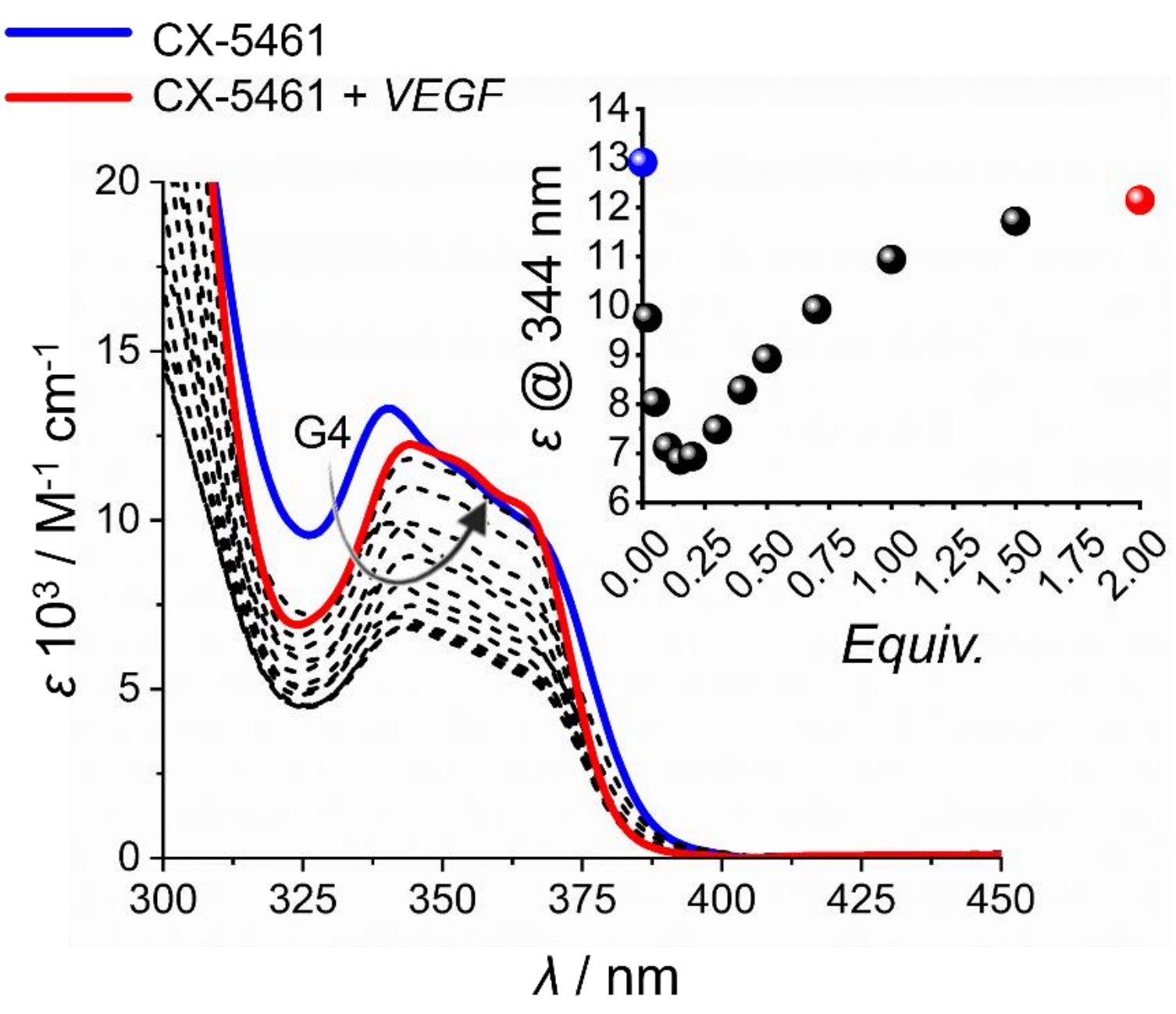

Figure S3. Absorption spectral changes of **CX-5461** (10 µM) upon titration with *VEGF* G4 DNA (0–20 µM) in Tris buffer (50 mM, pH 6.8, 100 mM KCl). Blue and red traces correspond to 0 and 2.0 equiv of *VEGF* G4 DNA, respectively; dashed lines represent intermediate concentrations. Inset: Changes in molar absorptivity at 344 nm.

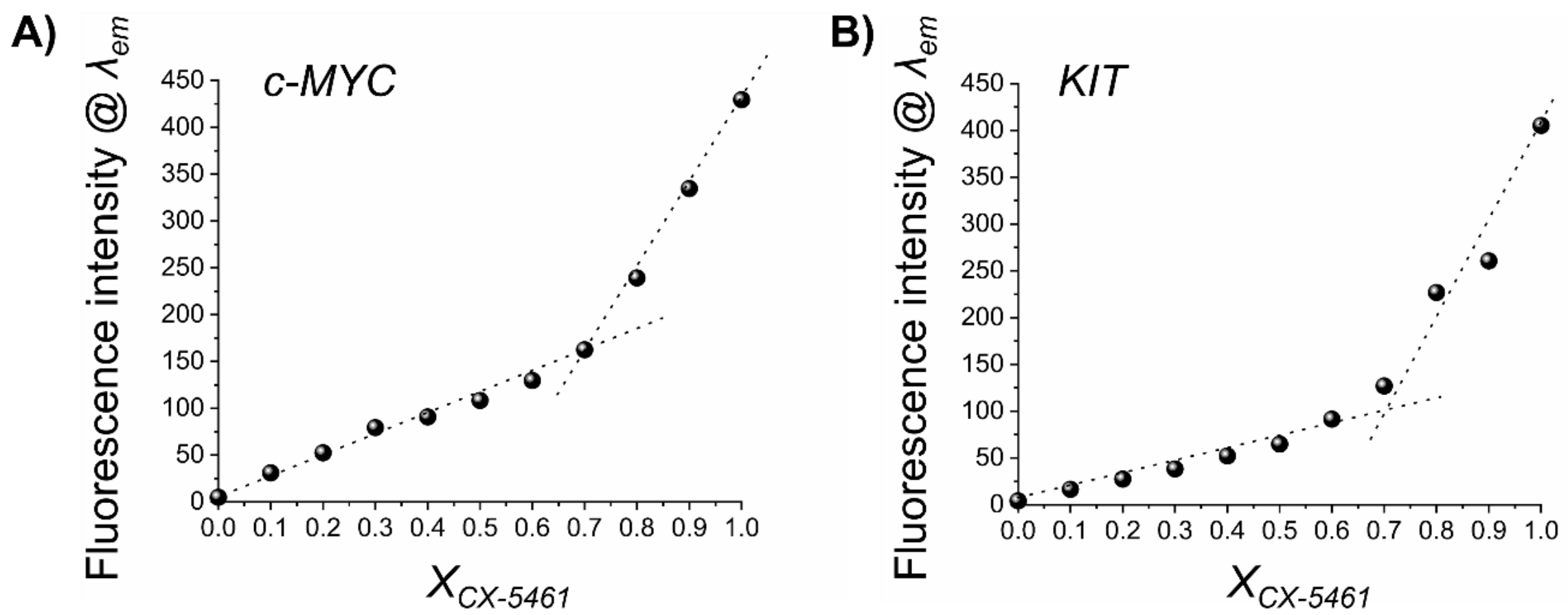

Figure S4. Fluorescence-based Job plots for **CX-5461** with *c-MYC* (**A**) and *KIT* (**B**) obtained by varying the mole fraction of **CX-5461** and G4 while keeping the total concentration constant at 10 µM. Fluorescence intensities were extracted at the corresponding emission maximum. Measurements were performed in Tris buffer (50 mM Tris, pH 6.8, 100 mM KCl) with excitation at λ_{exc} = 365 nm. Dashed lines indicate linear fits to the two regimes, whose intersection supports an apparent 2:1 **CX-5461** binding stoichiometry.

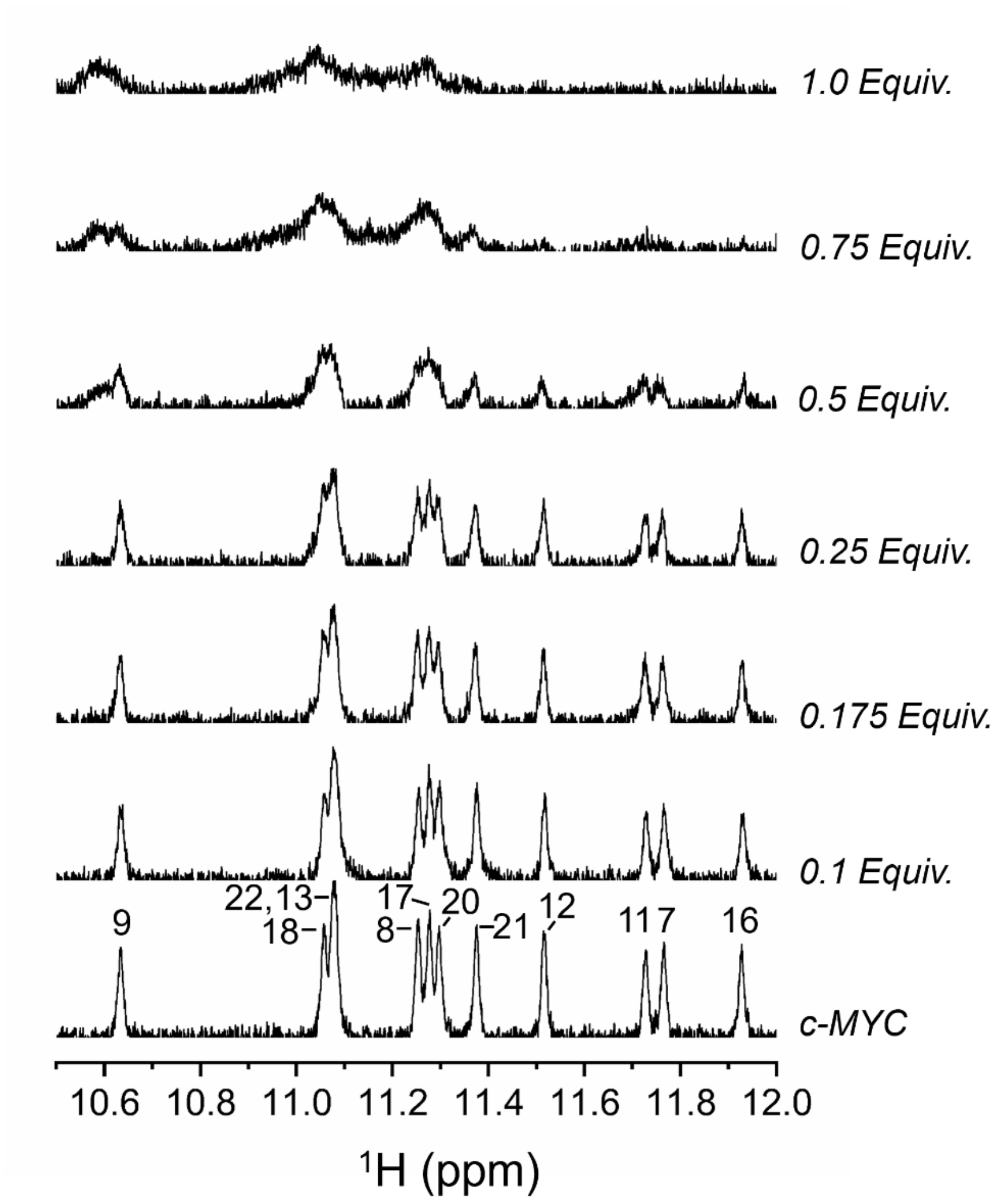

Figure S5. Imino proton region of the ^{1}H NMR spectra of *c-MYC* G4 recorded upon addition of increasing concentrations of **CX-5461**. Measurements were performed with *c-MYC* G4 at 200 µM and **CX-5461** at 0, 20, 35, 50, 100, 150, and 200 µM, corresponding to 0, 0.10, 0.175, 0.25, 0.50, 0.75, and 1.0 equivalents of **CX-5461** relative to G4. Spectra were acquired in 10 mM Tris buffer, pH 6.8, containing 70 mM KCl. Assigned guanine imino proton resonances of the unbound *c-MYC* G4 are labelled at the bottom of the spectrum.

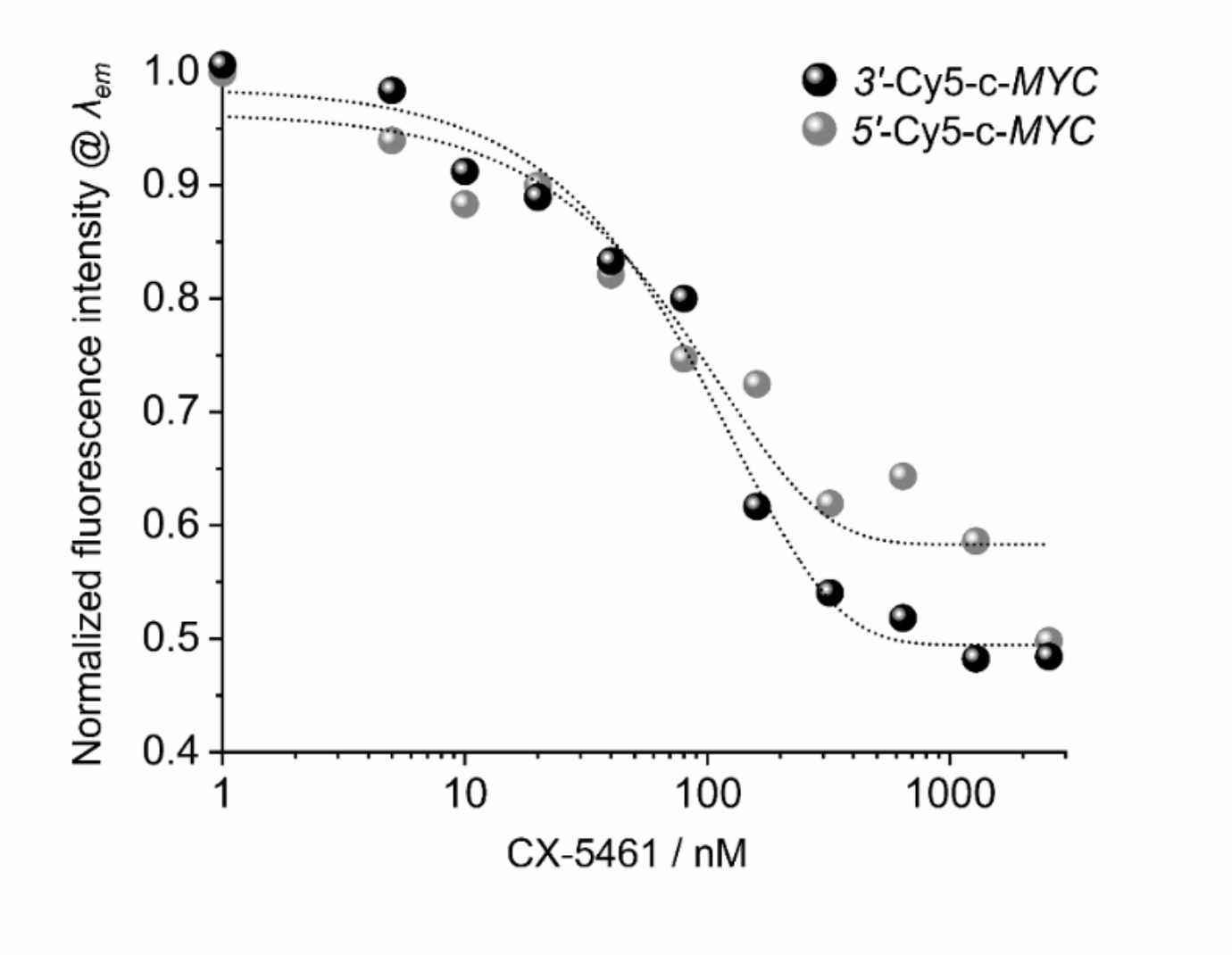

Figure S6. Normalized Cy5 fluorescence emission of *c-MYC* G4 (10 nM) labelled with Cy5 at either the *5′* or *3′* terminus upon titration with **CX-5461** up to 2560 nM. Fluorescence intensities were extracted at the emission maximum of each free labelled construct, λ_{em} = 666 nm for *5′*-Cy5-*c-MYC* and λ_{em} = 664 nm for *3′*-Cy5-*c-MYC*, and normalized to the corresponding signal in the absence of **CX-5461**, F/F_0. Measurements were performed with excitation at λ_{exc} = 645 nm. The **CX-5461** concentration is plotted on a logarithmic scale. Dashed lines are included to guide the eye.

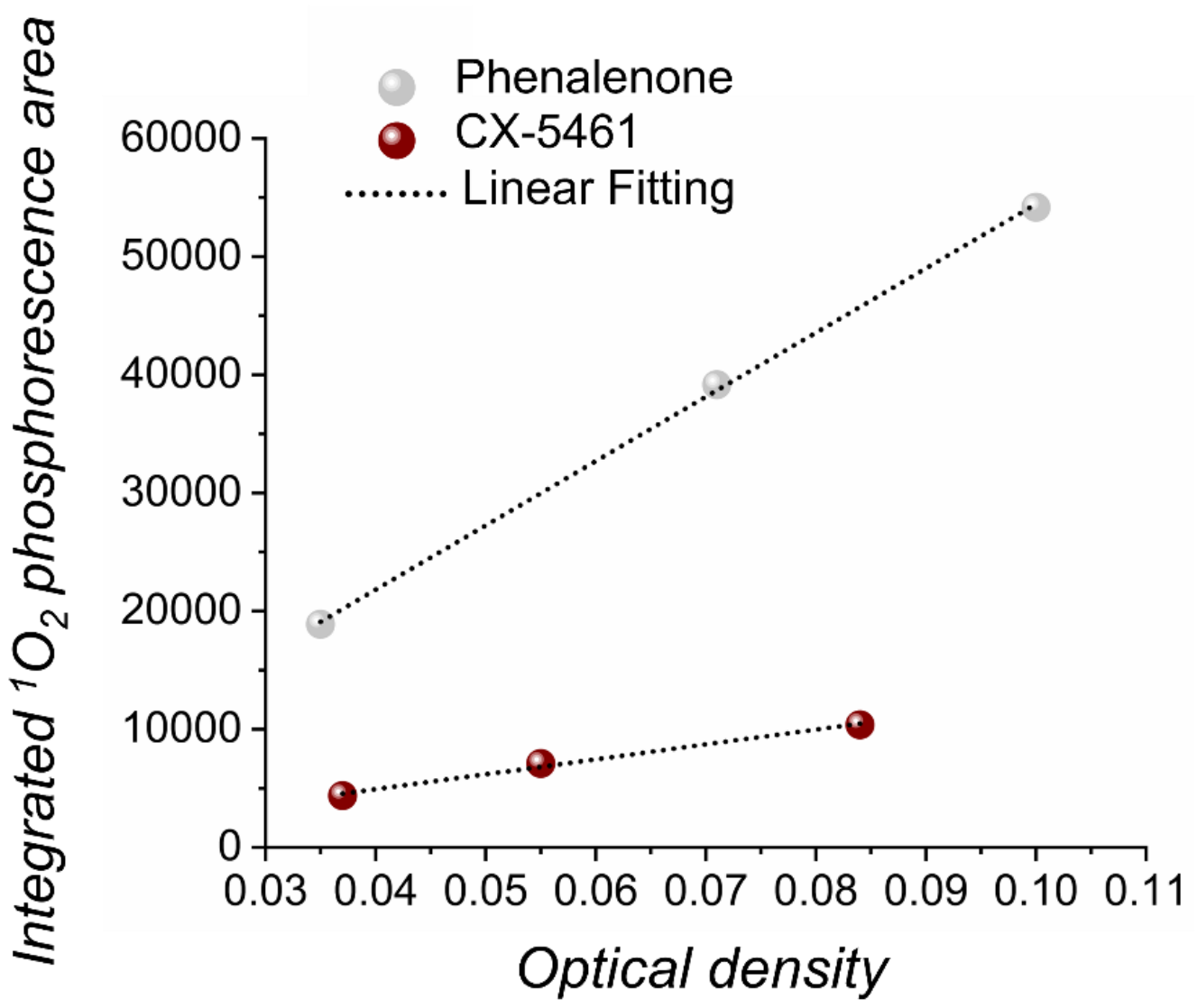

Figure S7. In situ quantification of $^1O_2$ generation by **CX-5461** and phenalenone based on NIR $^1O_2$ phosphorescence measurements under 365 nm irradiation in $CHCl_3$. The linear dependence of the integrated $^1O_2$ phosphorescence intensity on absorbed light dose enables comparison of $^1O_2$ production efficiencies and determination of Φ_Δ.

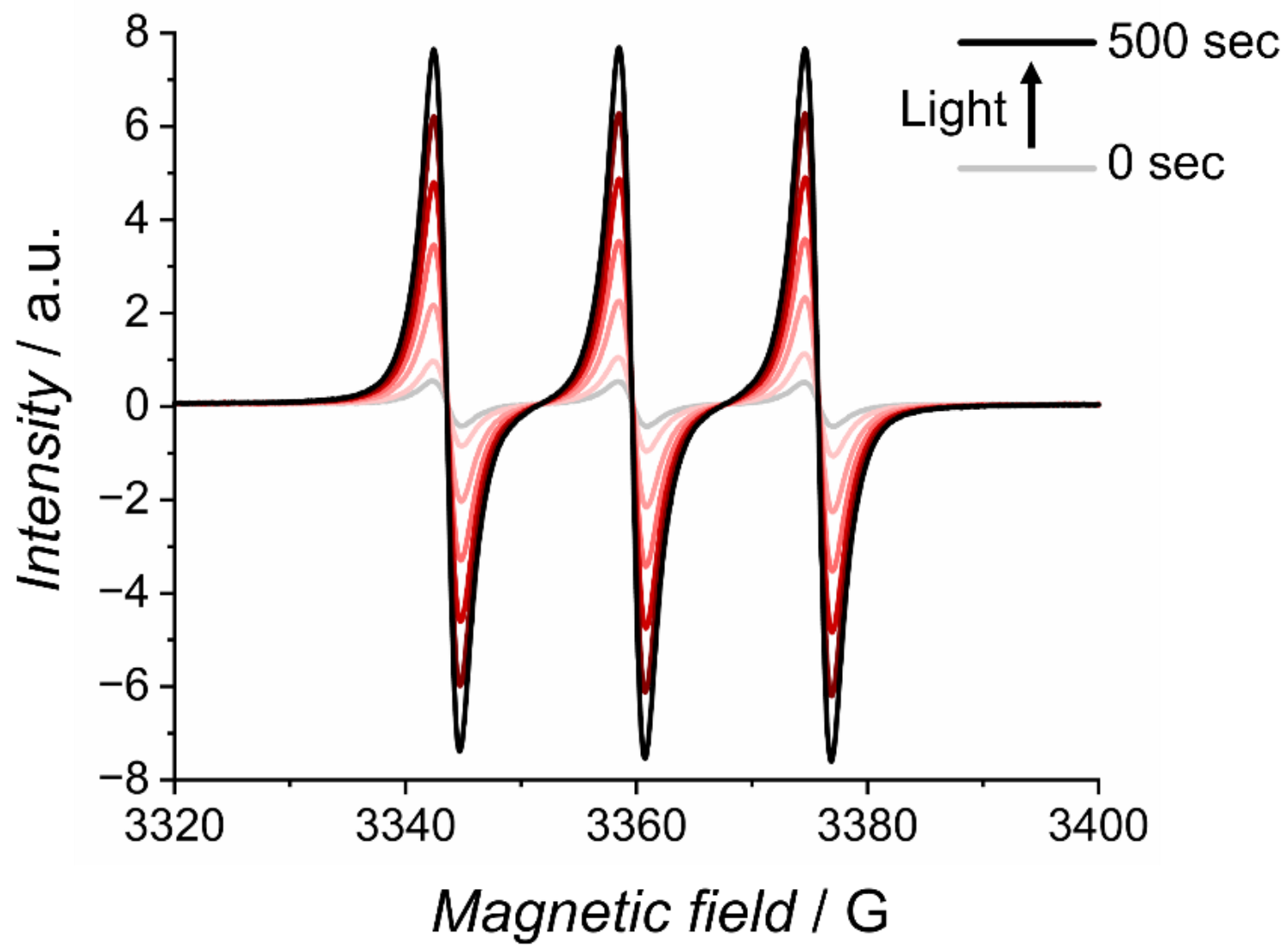

Figure S8. In situ monitoring of $^1O_2$ generation using reference compound phenalenone as a photosensitizer and TEMP as a spin-trapping agent under 365 nm irradiation in $CHCl_3$. The EPR spectra show the progressive formation of the TEMPO radical, indicating phenalenone-mediated $^1O_2$ production.

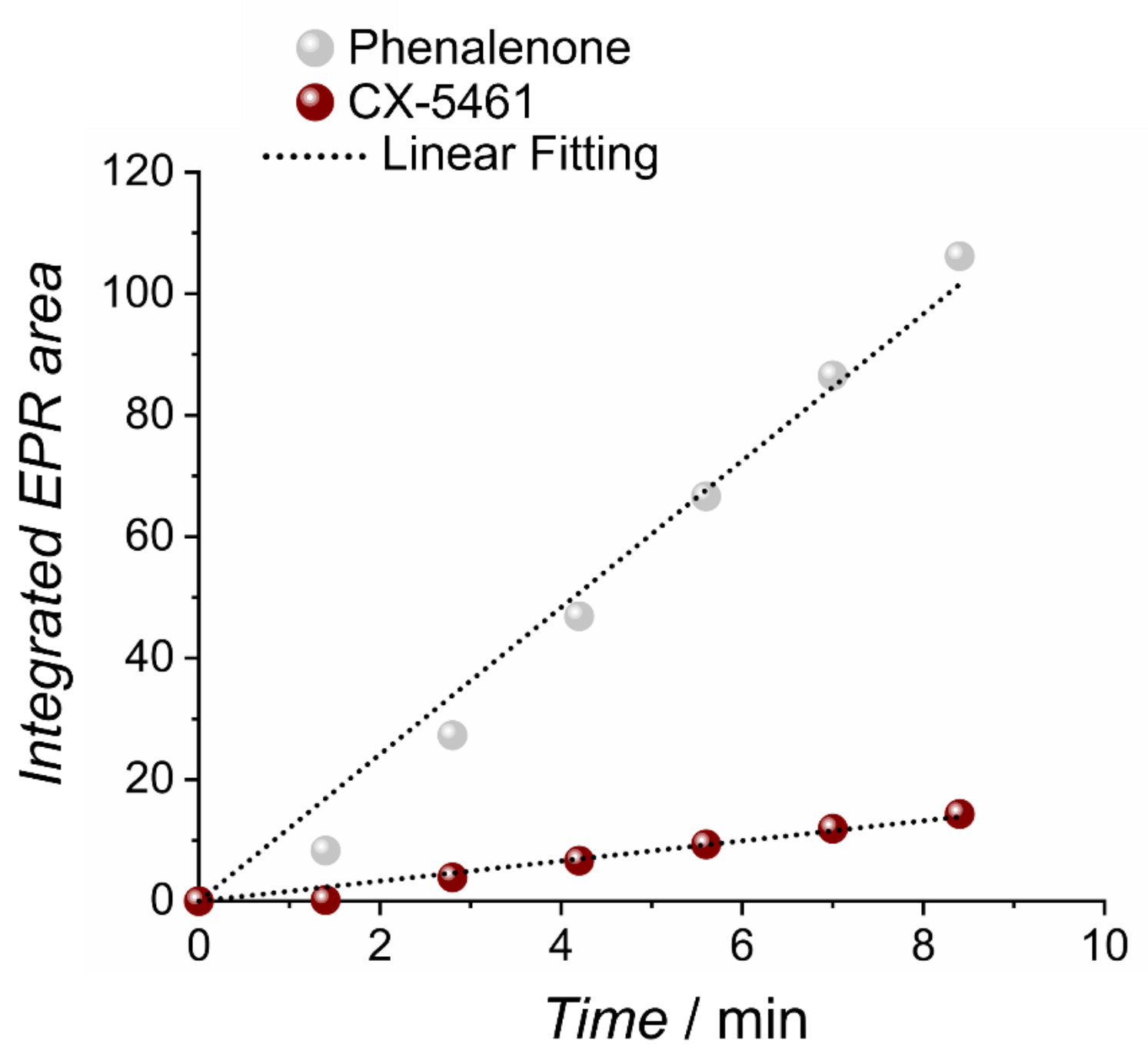

Figure S9. In situ monitoring of $^1O_2$ generation using TEMP under 365 nm irradiation in $CHCl_3$. The increase in TEMPO EPR signal intensity exhibits a linear dependence, confirming phenalenone- and **CX-5461**-mediated $^1O_2$ production and enabling quantification of the Φ_Δ.

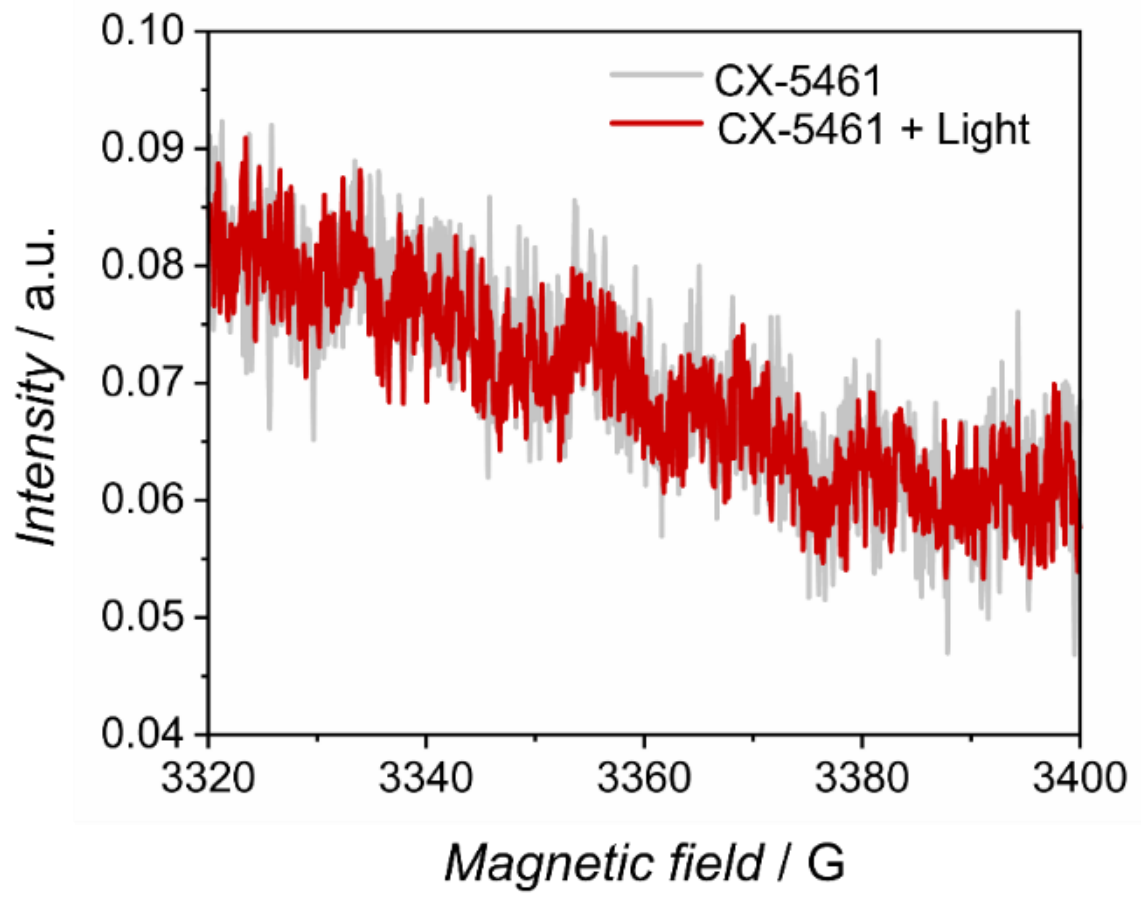

Figure S10. EPR spectra of **CX-5461** in DMSO obtained without irradiation (gray) and under 365 nm light irradiation (red), in the absence of a spin trap. Irradiation does not produce a resolved radical signature.

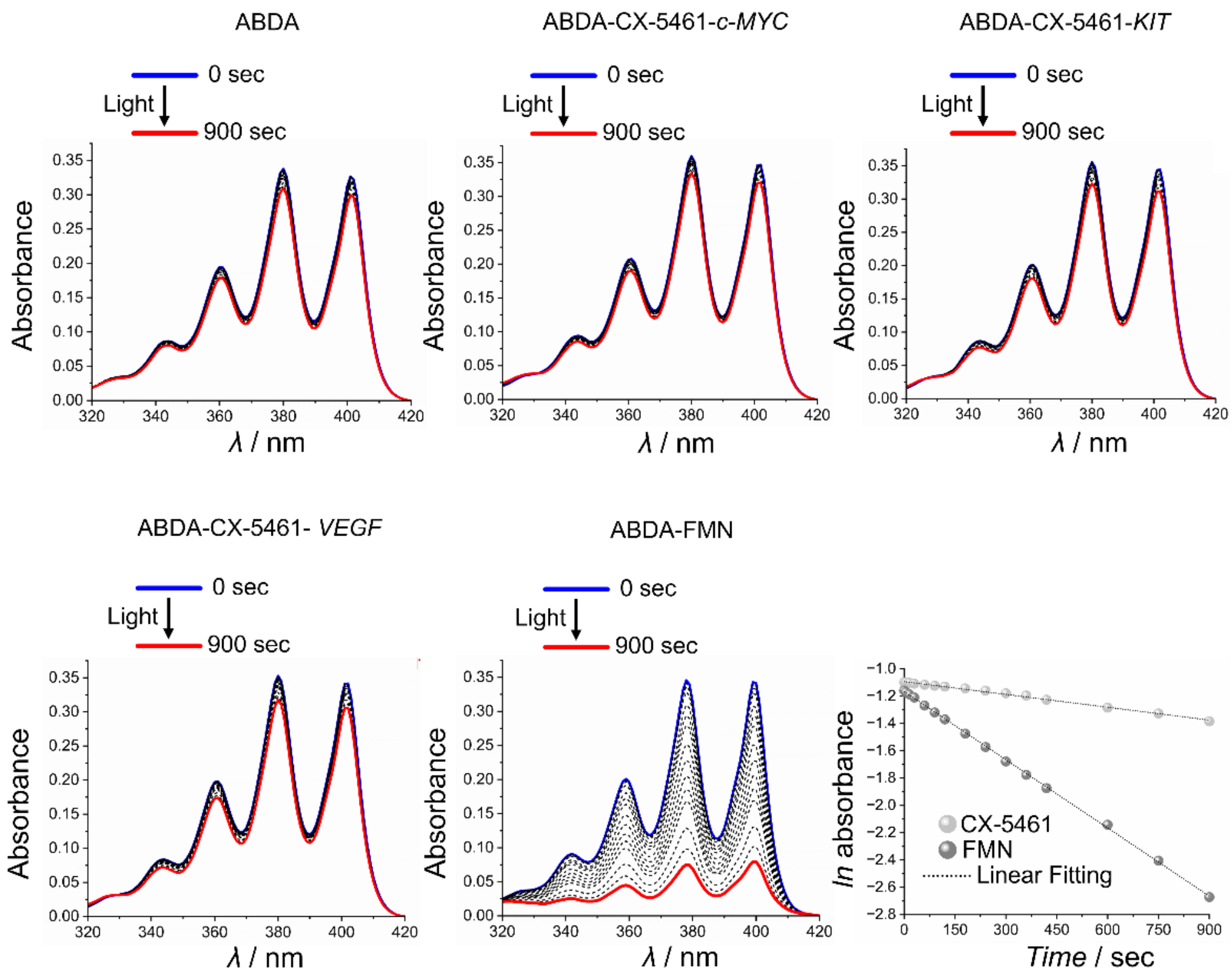

Figure S11. Monitoring of $^1O_2$ production via ABDA (30 µM) photobleaching. **CX-5461** (15 µM) bound to *c-MYC*, *KIT*, or *VEGF* G4 DNA (15 µM) in Tris–phosphate buffer (50 mM, pH 6.8, 100 mM KCl). ABDA decay was followed at 380 nm during 365 ± 10 nm irradiation. FMN in water (Φ_Δ = 0.51) served as the reference photosensitizer. Blue and red traces correspond to 0 and 900 s of irradiation, respectively; dashed lines represent intermediate time points. Pseudo-first-order plots are presented as ln absorbance vs time.

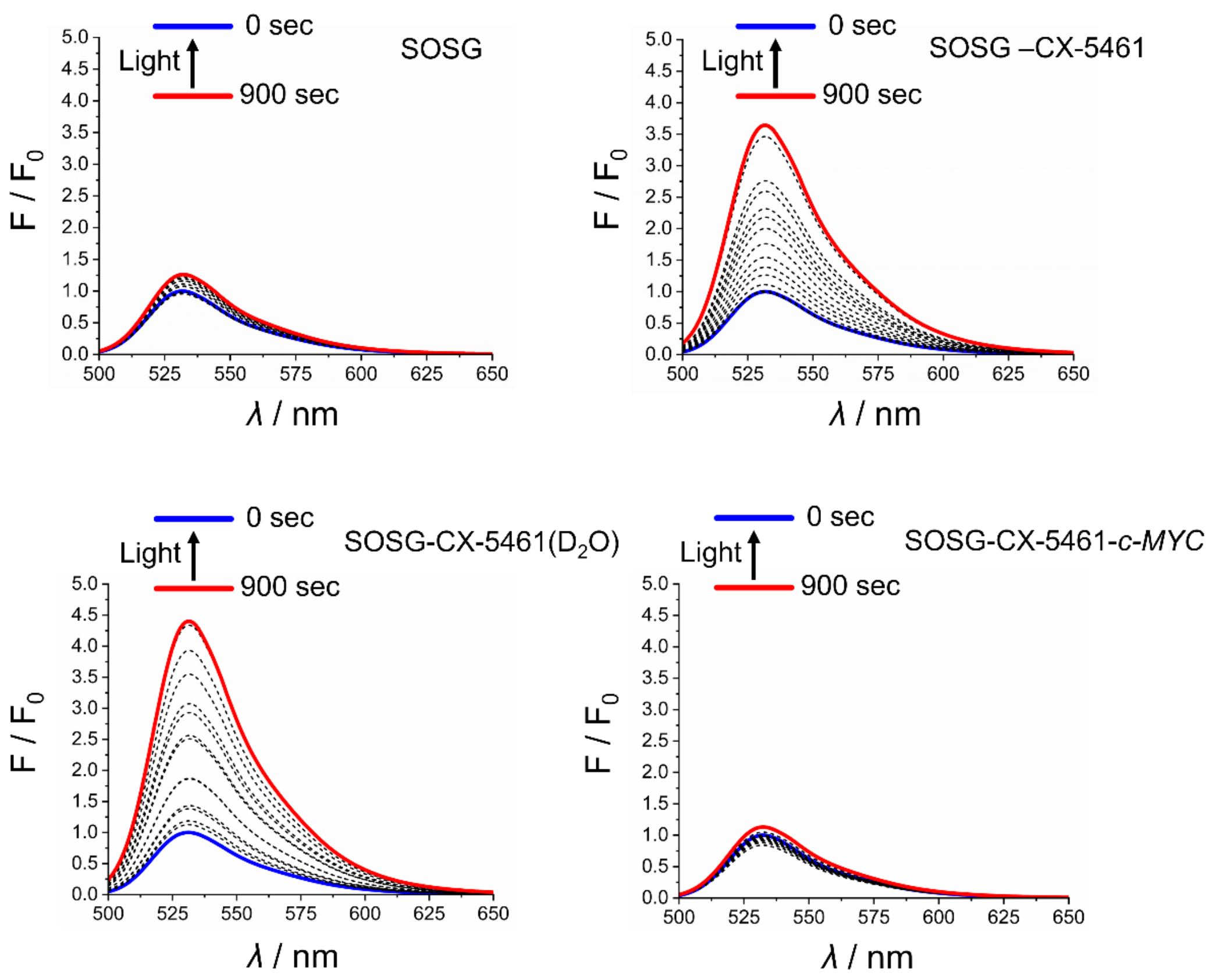

Figure S12. Monitoring of $^1O_2$ generation by SOSG (5 µM) fluorescence enhancement. Emission spectral changes of SOSG upon 365 nm irradiation in the presence of **CX-5461** (15 µM), either free in solution or in complex with *c-MYC* G4 DNA (15 µM), recorded in Tris–phosphate buffer (50 mM, pH 6.8, 100 mM KCl). Measurements for **CX-5461** were additionally performed in D_2O-containing Tris–phosphate buffer under identical conditions. Blue and red traces correspond to 0 and 900 s of irradiation, respectively; dashed lines represent intermediate time points.

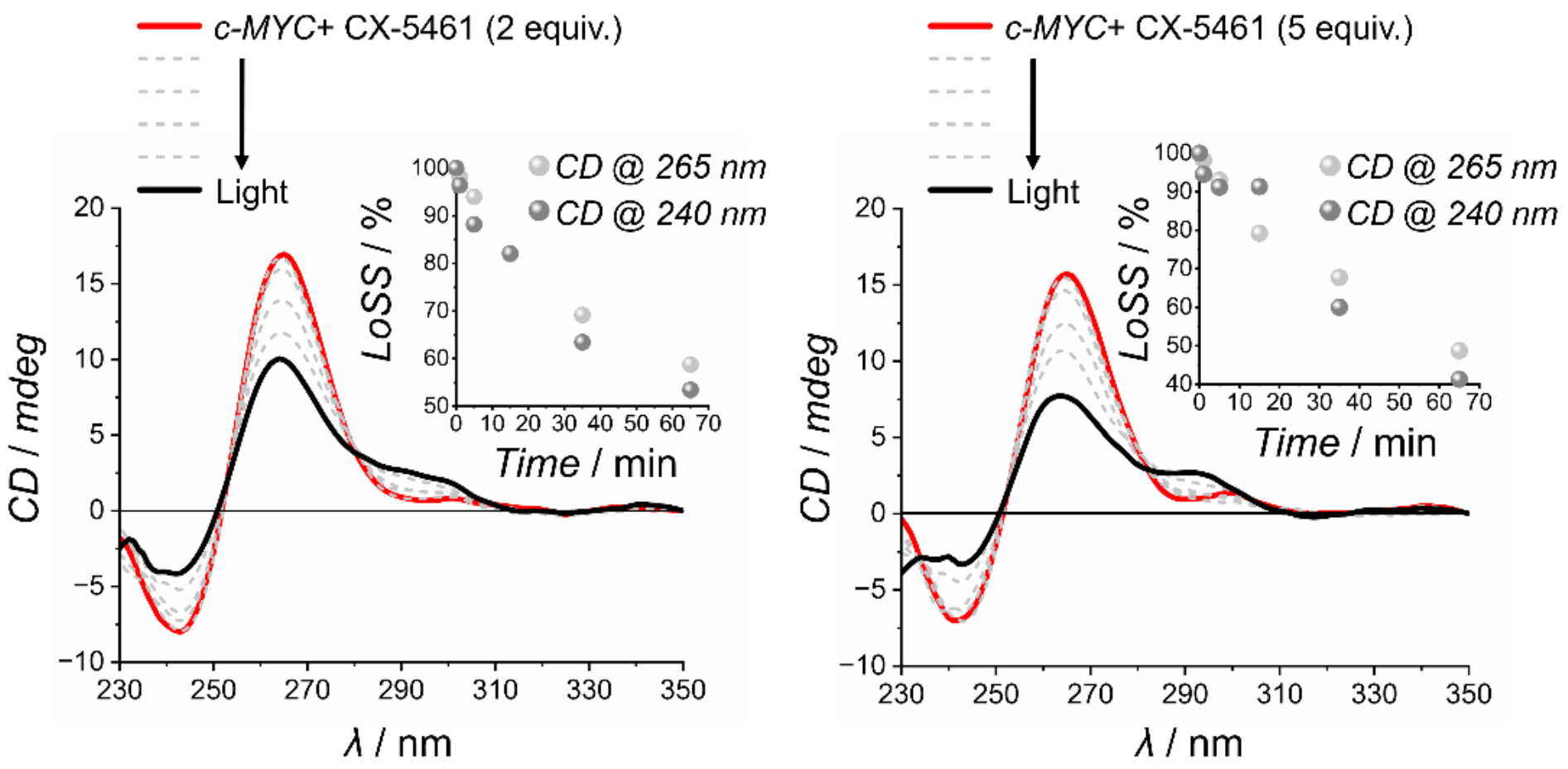

Figure S13. CD spectra of the *c-MYC* G4 recorded in the absence and presence of **CX-5461** under dark conditions or upon 365 nm irradiation. Measurements were performed using *c-MYC* G4 (2 μM) and CX-5461 (4 or 10 μM) in Tris buffer (10 mM, pH 6.8) containing 100 mM KCl. Inset: loss of secondary structure (LoSS) monitored at 240 and 265 nm.

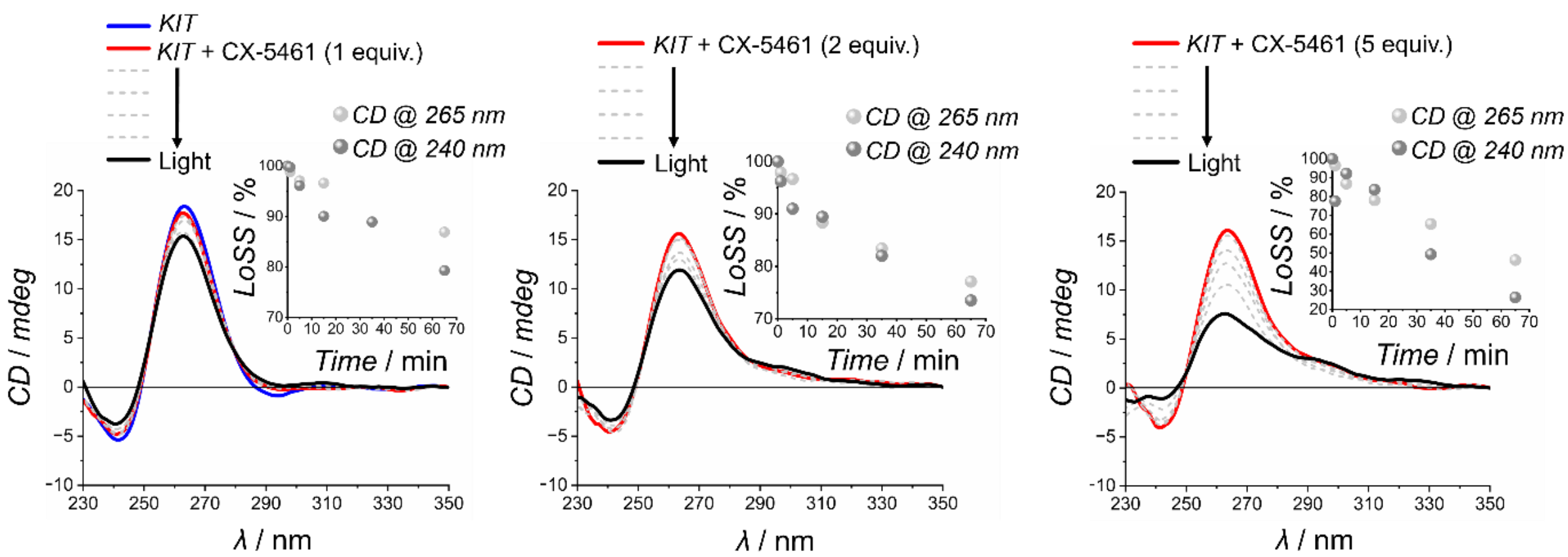

Figure S14. CD spectra of the *KIT* G4 recorded in the absence and presence of **CX-5461** under dark conditions or upon 365 nm irradiation. Measurements were performed using *KIT* G4 (2 μM) and **CX-5461** (0, 2, 4 or 10 μM) in Tris buffer (10 mM, pH 6.8) containing 100 mM KCl. Inset: loss of secondary structure (LoSS) monitored at 240 and 265 nm.

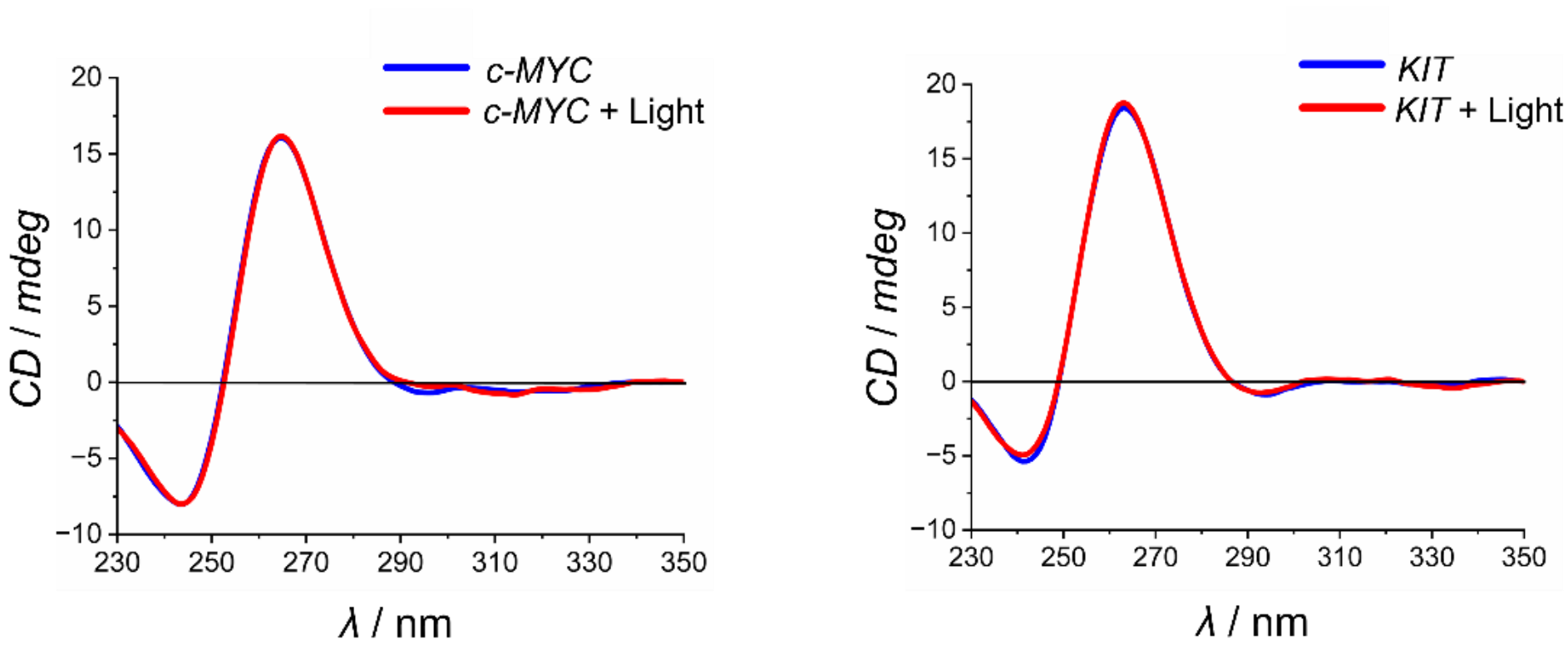

Figure S15. CD spectra of the *c-MYC* and *KIT* G4 recorded under dark conditions or upon 365 nm irradiation for 65 minutes. Measurements were performed using *c-MYC* or *KIT* G4 (2 µM) in Tris buffer (10 mM, pH 6.8) containing 100 mM KCl.

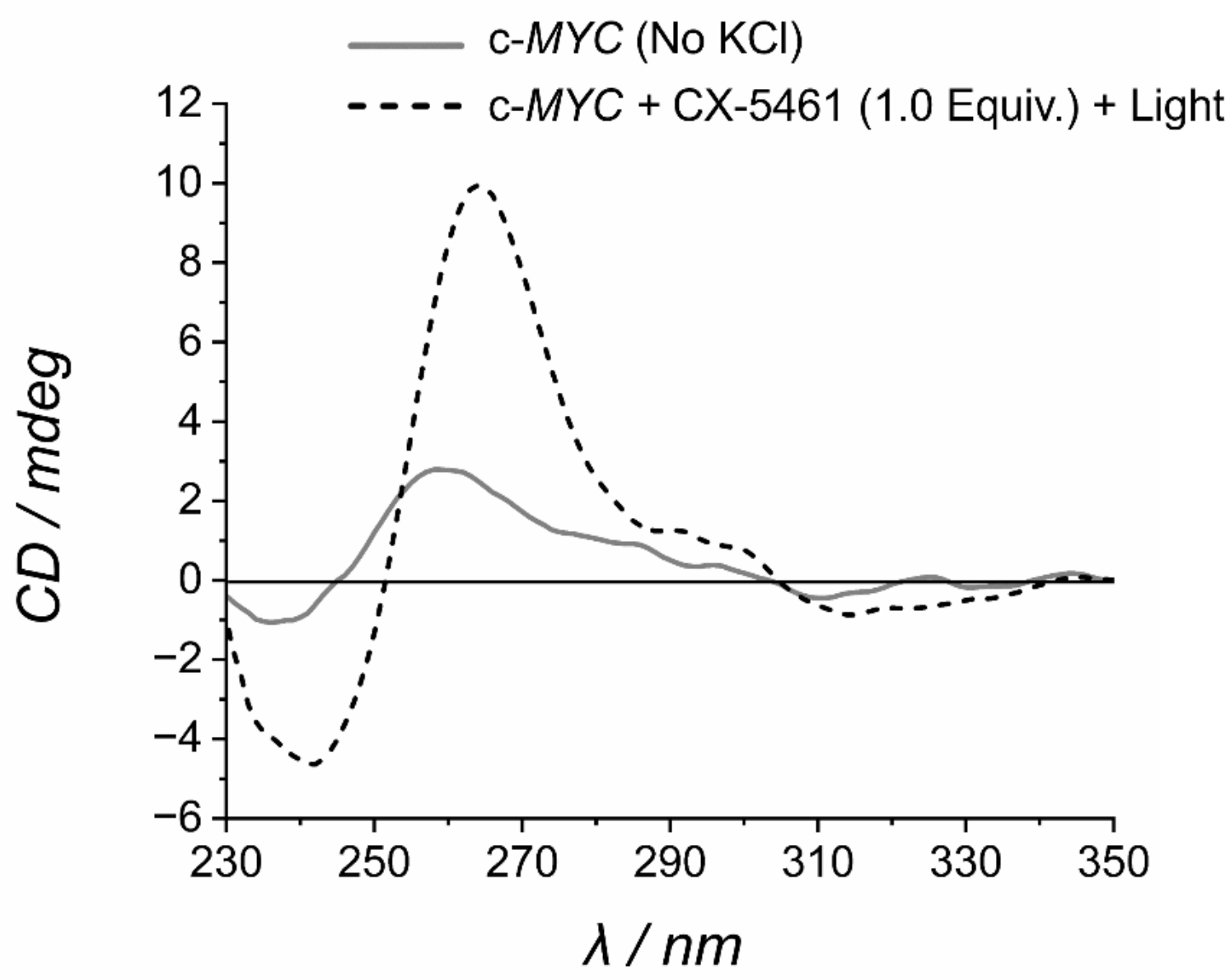

Figure S16. CD spectra of the irradiated *c-MYC* complex (black dashed line) and unfolded *c-MYC* control (solid gray line). The complex contained *c-MYC* and **CX-5461** at 2 µM each in 10 mM Tris buffer, pH 6.8, with 100 mM KCl, and was irradiated for 65 min at 365 nm. The unfolded *c-MYC* control was recorded at 2 µM DNA in 10 mM Tris buffer, pH 6.8, without KCl.

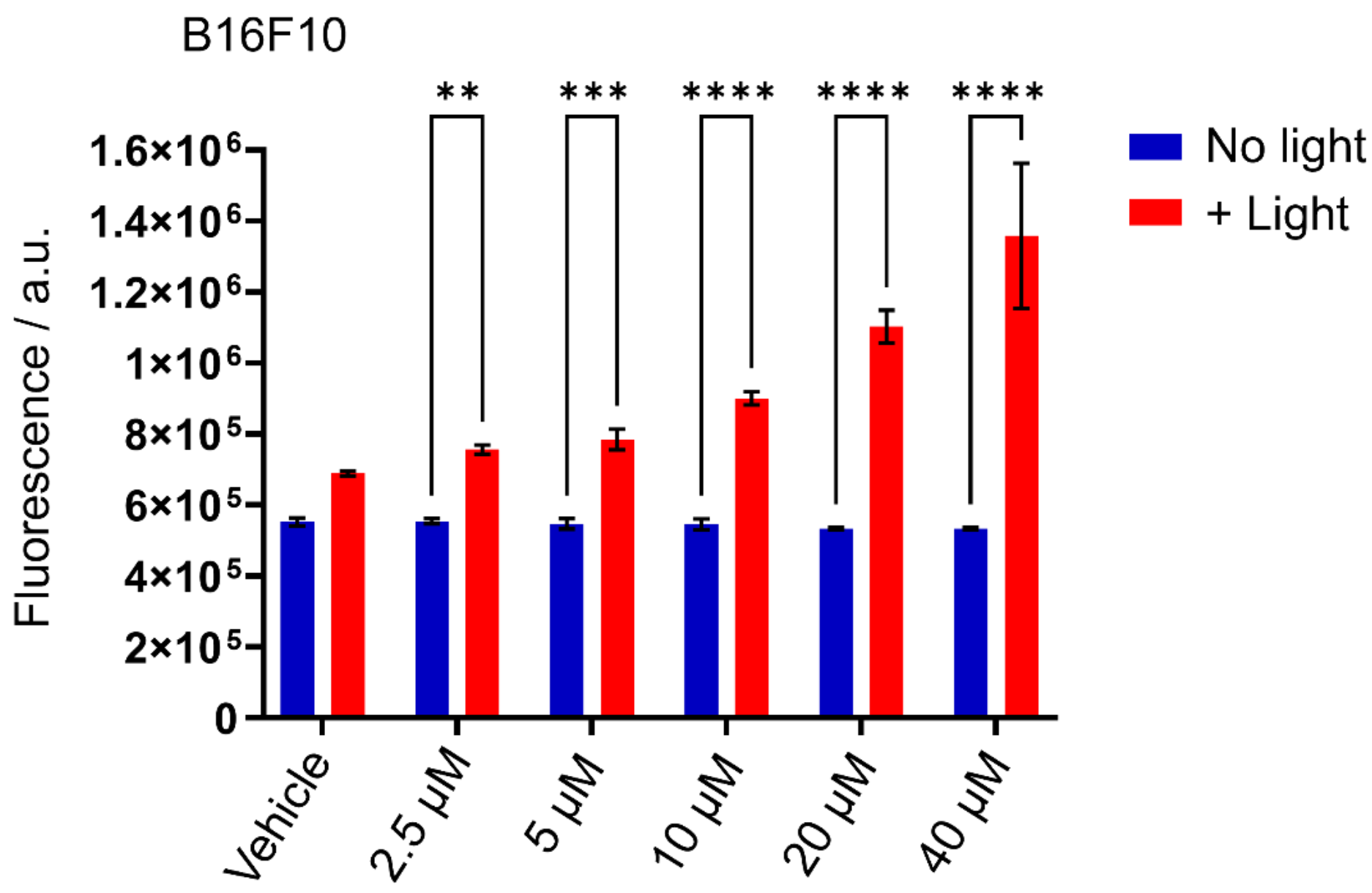

Figure S17. Intracellular ROS generation in B16F10 cells measured using H_2DCFDA following **CX-5461** treatment under dark and irradiated conditions. Error bars indicate mean ± SD (N = 3). Statistical analysis was performed using a two-way ANOVA; p values are indicated (**$p < 0.01$; ***$p < 0.001$; ****$p < 0.0001$).

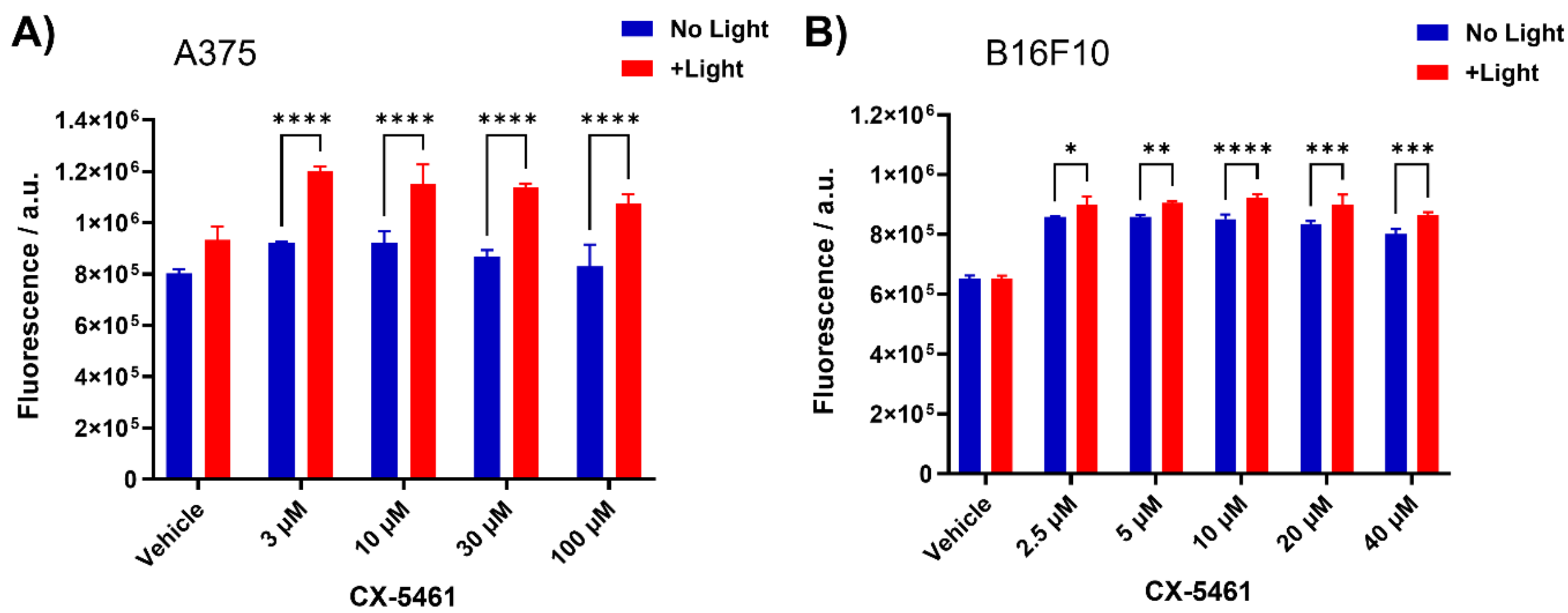

Figure S18. Intracellular ROS generation in A375 and B16F10 cells measured using DHR-123 following **CX-5461** treatment under dark and irradiated conditions. Error bars indicate mean ± SD (N = 3). Statistical analysis was performed using a two-way ANOVA; p values are indicated (*$p < 0.05$; **$p < 0.01$; ***$p < 0.001$; ****$p < 0.0001$).

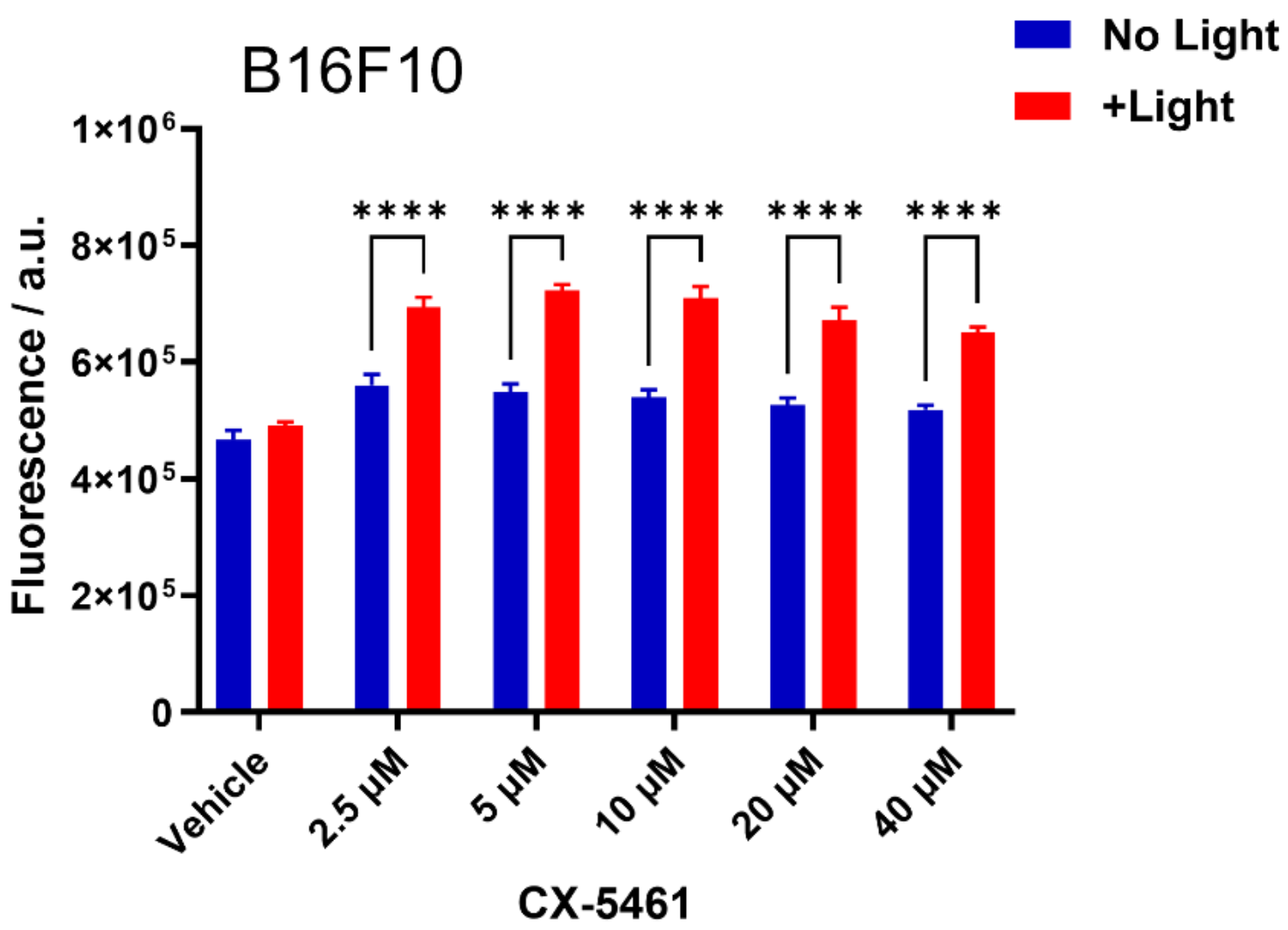

Figure S19. Intracellular ROS generation in B16F10 cells measured using HPF following **CX-5461** treatment under dark and irradiated conditions. Error bars indicate mean ± SD (N = 3). Statistical analysis was performed using a two-way ANOVA; p values are indicated (****p < 0.0001).

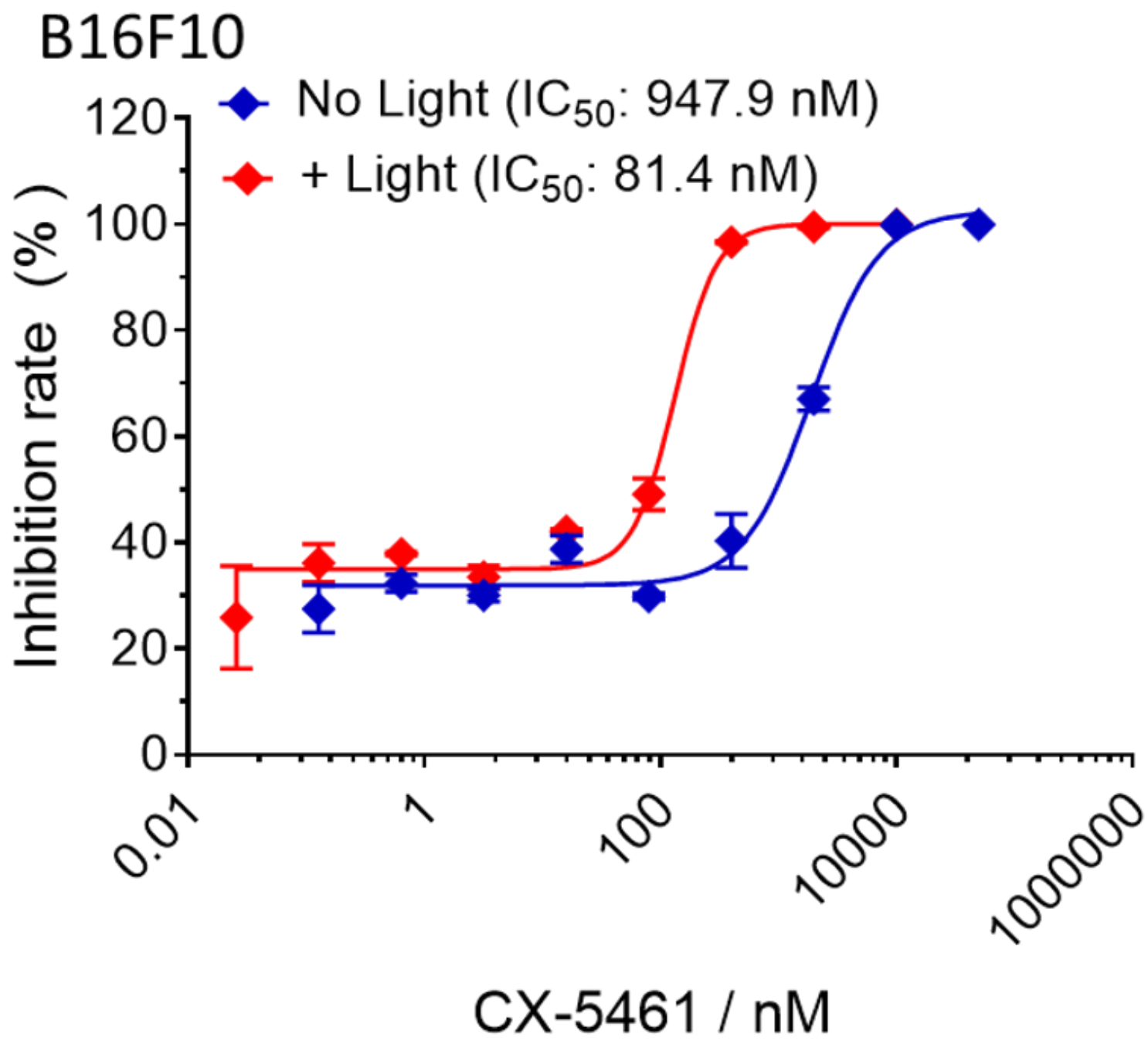

Figure S20. (Photo)cytotoxicity of **CX-5461** in B16F10 cells upon 365 nm irradiation (7 seconds, 100 mJ cm^{-2}).

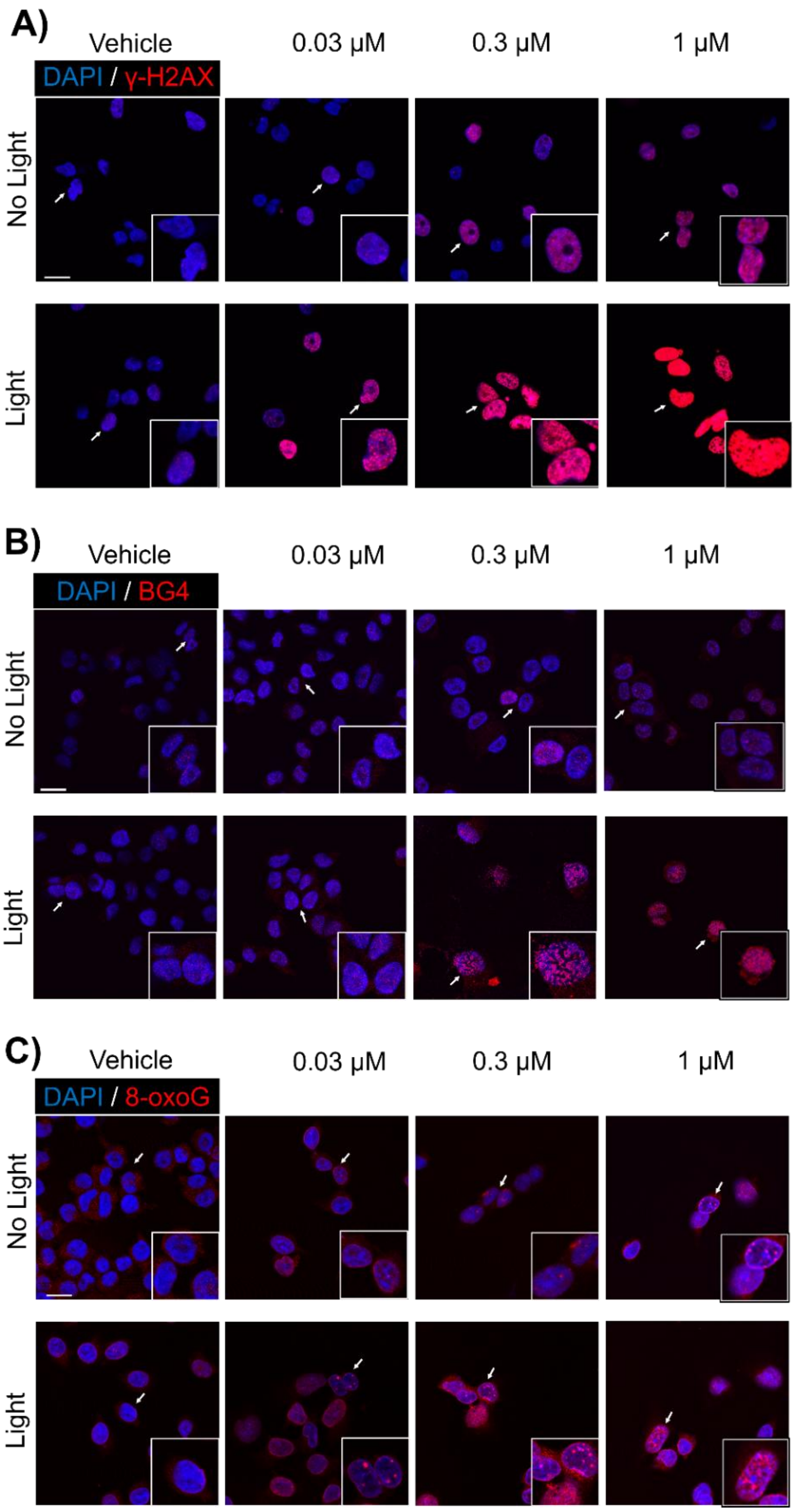

Figure S21. Representative immunofluorescence images showing γ-H2AX foci (A), nuclear G4 structures detected by BG4 (B), and 8-oxoG accumulation (C) in A375 cells treated with **CX-5461** under dark or light conditions (365 nm, 100 mJ cm^{-2}, for 7 seconds). Nuclei were counterstained with DAPI. Scale bar: 20 μm.

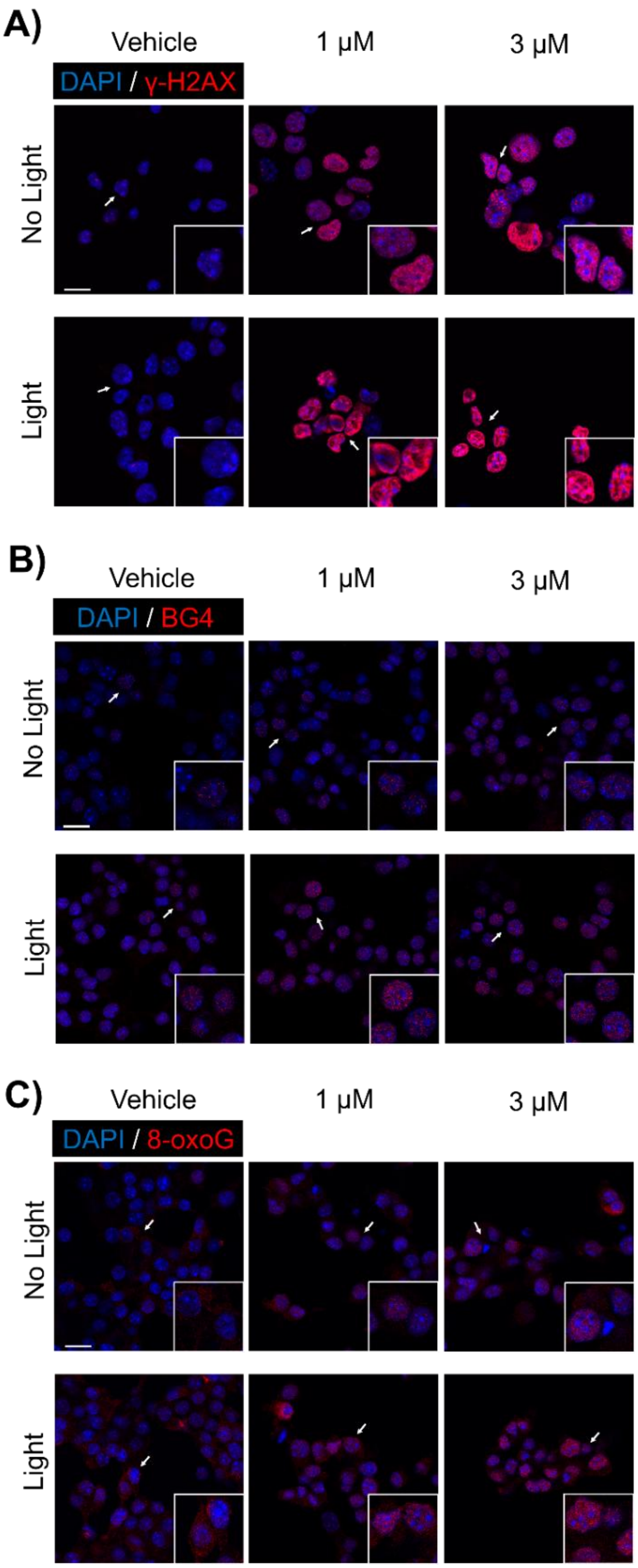

Figure S22. Representative immunofluorescence images showing γ-H2AX foci (A), nuclear G4 structures detected by BG4 (B), and 8-oxoG accumulation (C) in B16F10 cells treated with **CX-5461** under dark or light conditions (365 nm, 100 mJ cm^{-2}, for 7 seconds). Nuclei were counterstained with DAPI. Scale bar: 20 μm.

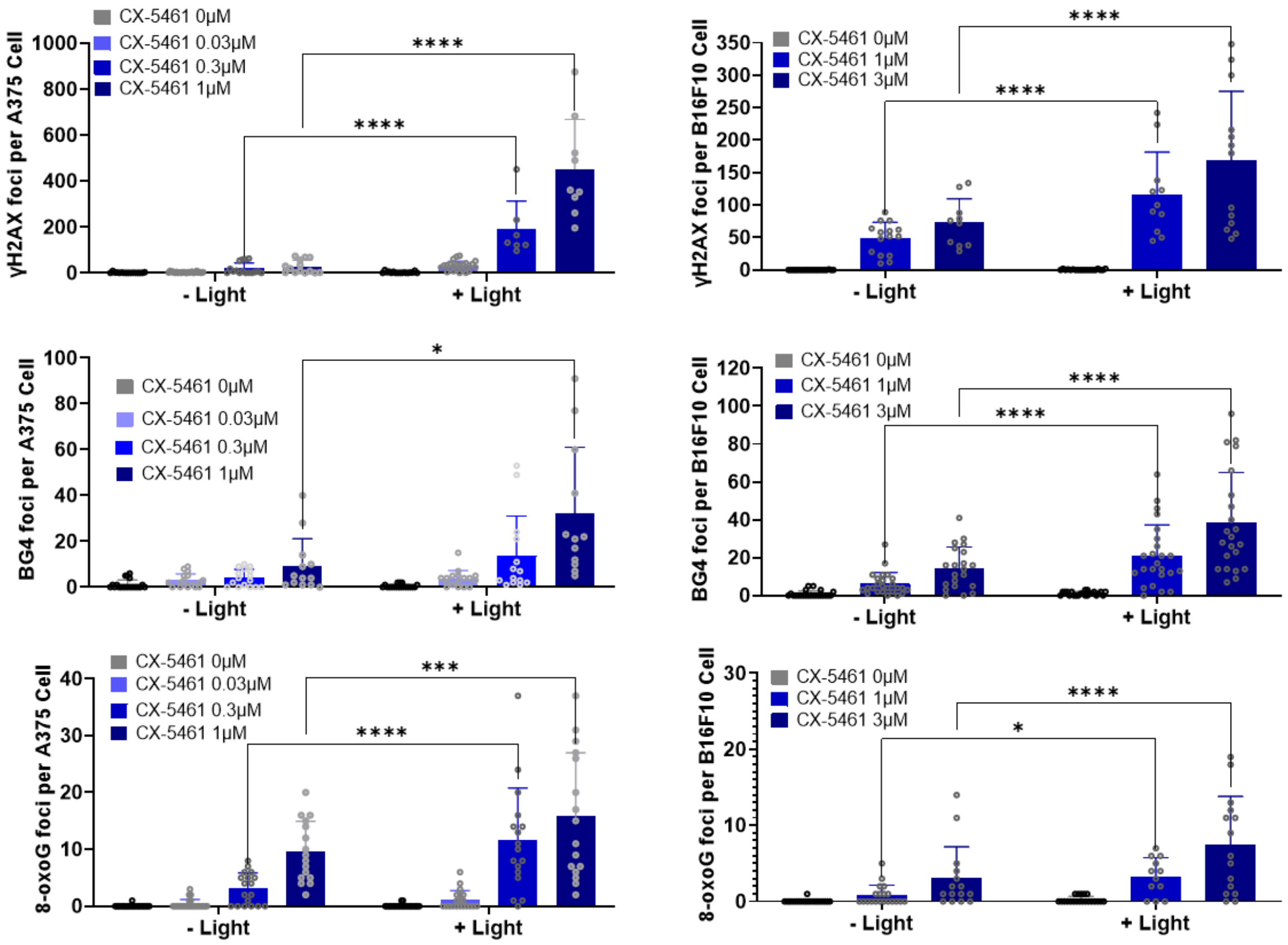

Figure S23. Signal quantification from immunofluorescence images showing γ-H2AX foci, nuclear G4 structures detected by BG4, and 8-oxoG accumulation in A375 and B16F10 cells treated with **CX-5461** under dark or irradiated conditions. Data represent populations of individual cells. Error bars indicate mean ± SD. Statistical analysis was performed using two-way ANOVA; p values are indicated (*p < 0.05; ***p < 0.001; ****p < 0.0001).

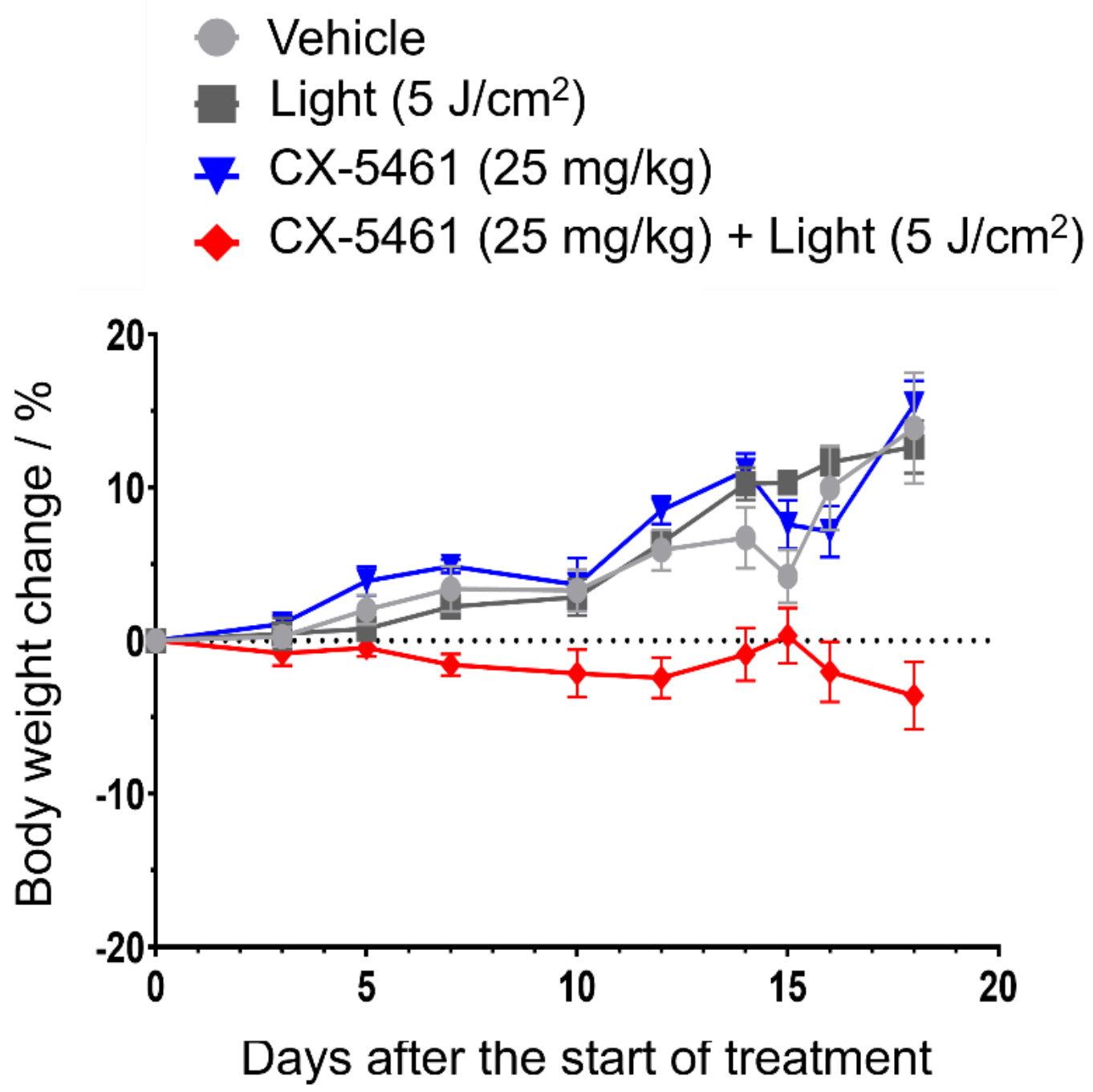

Figure S24. Body weight of CT26 tumor-bearing mice during treatment. Error bars represent mean ± SD (*N* = 5).

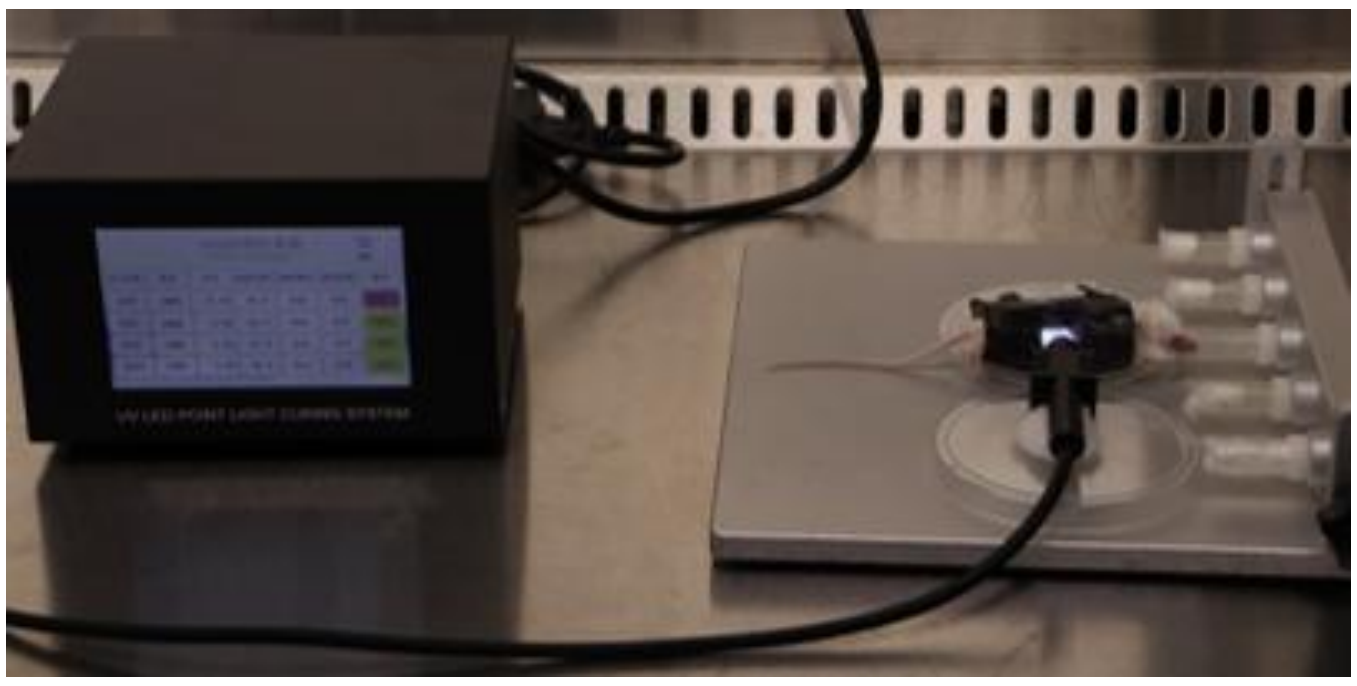

Figure S25. Image of the light delivery system used to photoexcite **CX-5461** in tumor-bearing mice.

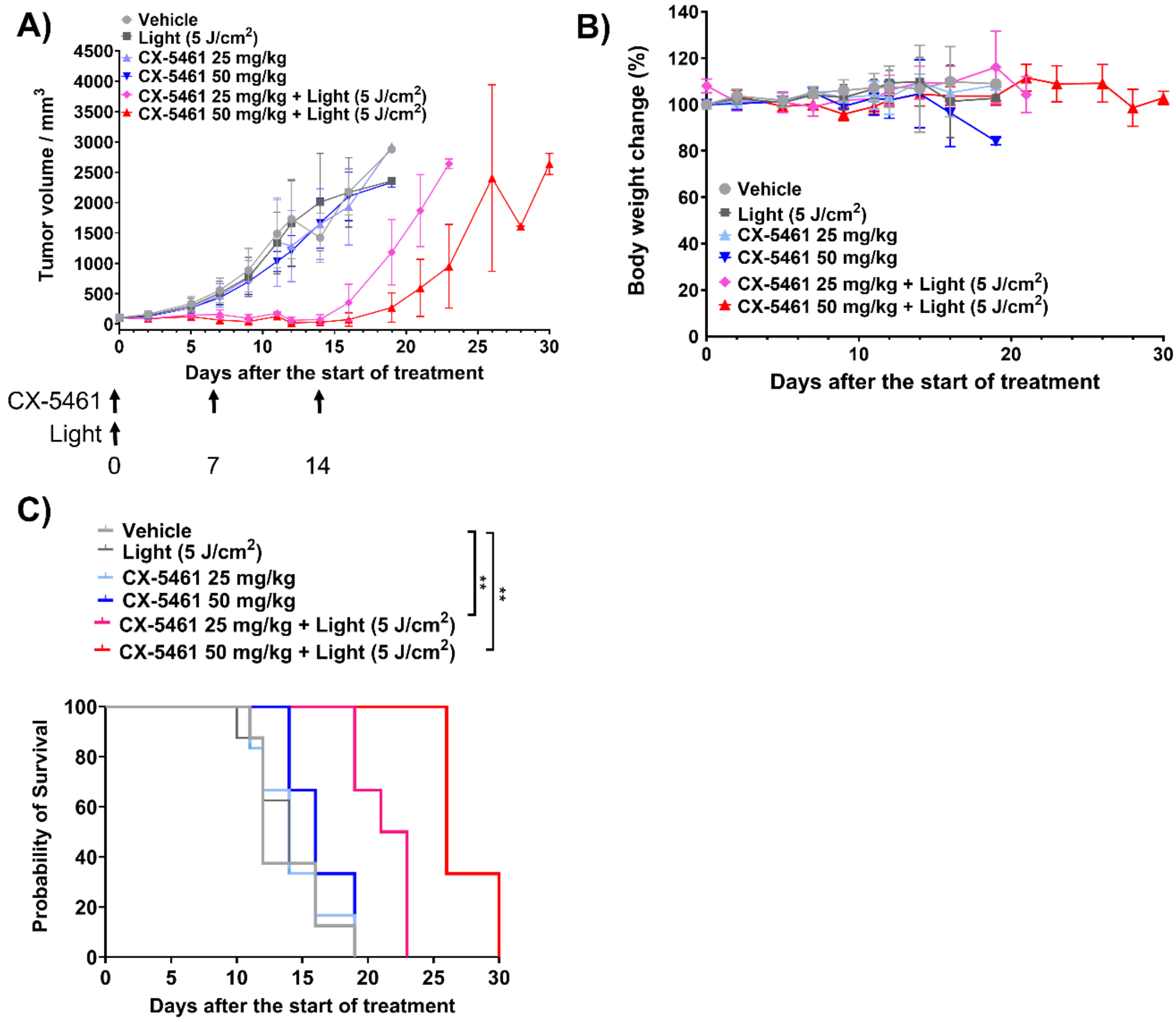

Figure S26. A) Antitumor efficacy of **CX-5461** in B16F10 tumor-bearing mice under nonirradiated and irradiated conditions. **B)** Body weight of B16F10 tumor-bearing mice during treatment. **C)** Kaplan–Meier survival curves of mice following **CX-5461** treatment. Error bars represent mean ± SD (N = 5). Statistical analysis was performed using log-rank test; p values are indicated (**$p < 0.01$).

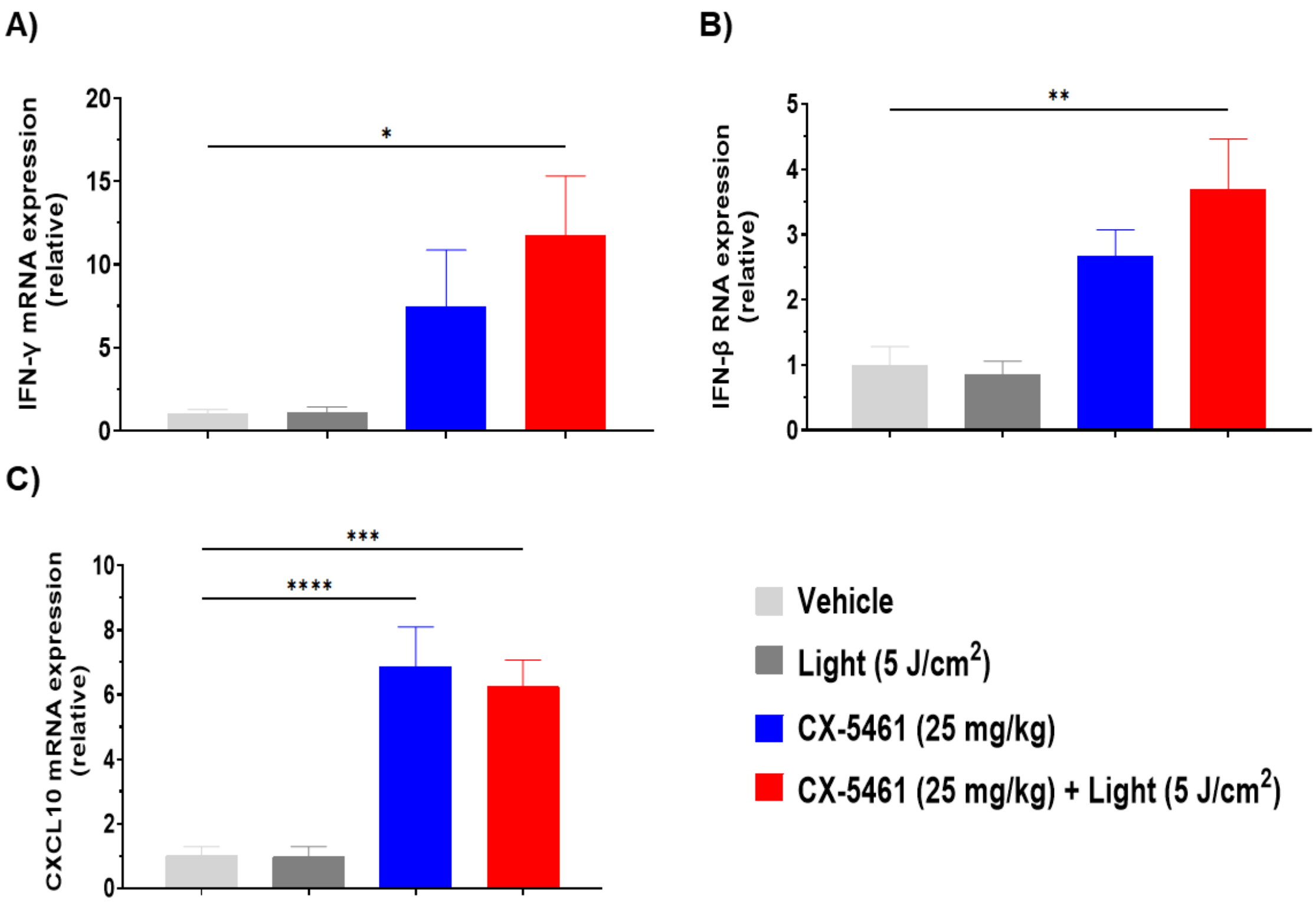

Figure S27. CT26 tumors were collected on Day 18, and the expression levels of **A)** IFN-γ, **B)** IFN-β, and **C)** CXCL10 in these tumors were measured using RT-PCR. The 18S rRNA gene was used as the internal control. Error bars represent mean ± SD (N = 5). Statistical analysis was performed using one-way ANOVA; p values are indicated (*p < 0.05; **p < 0.01; ***p < 0.001; ****p < 0.0001).

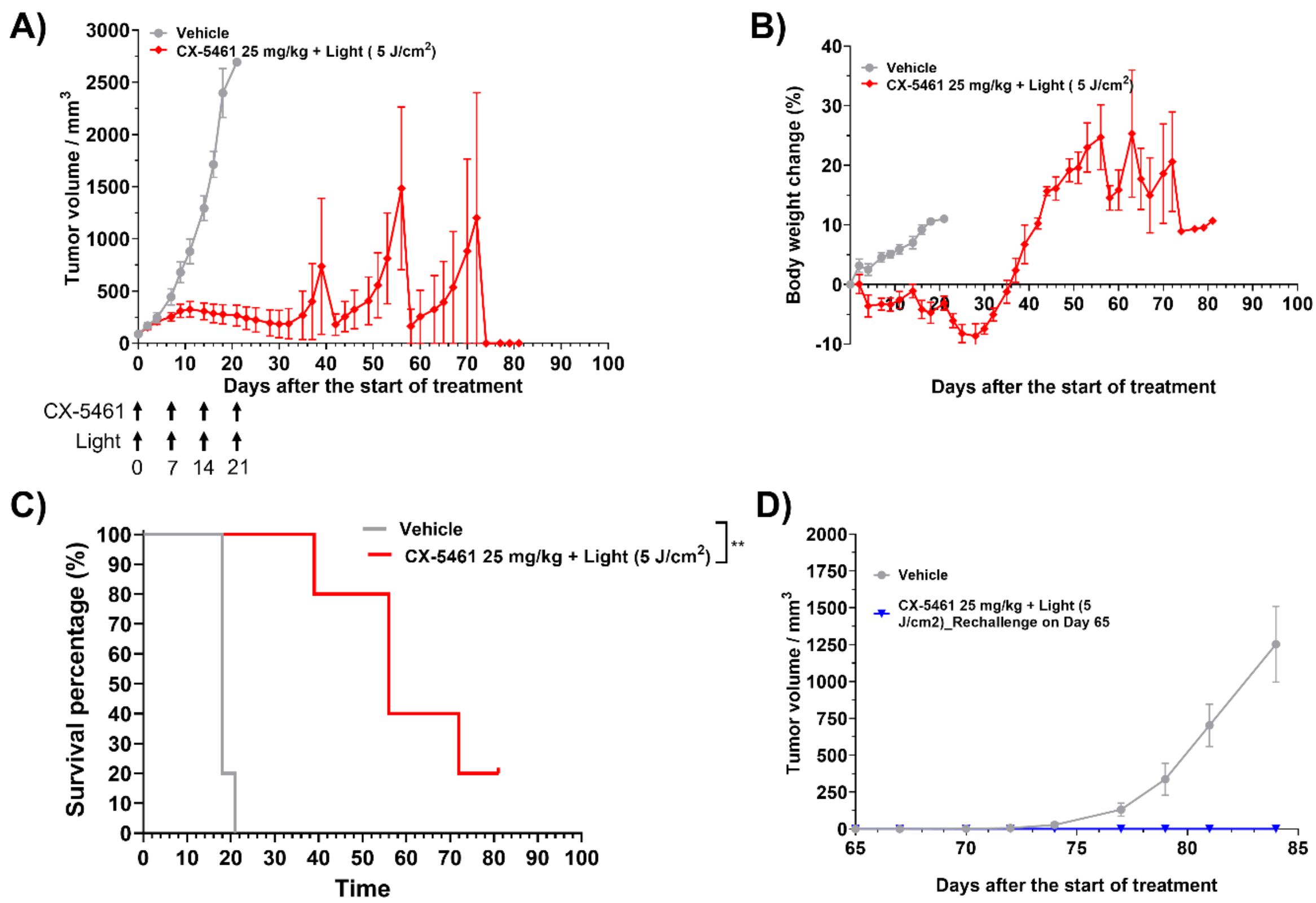

Figure S28. CT26 tumor rechallenge following complete regression induced by **CX-5461** photoactivation. **A)** Tumor growth curves from the original CT26 efficacy cohort treated weekly with vehicle alone or **CX-5461** plus localized 365 nm irradiation. One out of five mice in the **CX-5461** + light group achieved complete tumor regression. Error bars represent mean ± SD (*N* = 5). **B)** Body weight monitoring during the original CT26 treatment experiment. **C)** Kaplan–Meier survival analysis from the original CT26 treatment experiment. Statistical analysis was performed using Long-rank test; *p* value is indicated (***p* < 0.01). **D)** Tumor rechallenge experiment. The complete responder from the **CX-5461** + light group (*N* = 1) was rechallenged with CT26 cells after full tumor regression and showed no tumor regrowth, whereas naïve BALB/c control mice (*N* = 3) developed tumors. The rechallenge experiment was performed using the single available complete responder; therefore, this result is interpreted as evidence consistent with protective antitumor immune memory rather than definitive proof.

References

1. Haddach, M., Schwaebe, M.K., Michaux, J., Nagasawa, J., O'Brien, S.E., Whitten, J.P., Pierre, F., Kerdoncuff, P., Darjania, L., Stansfield, R. *et al*. (2012) Discovery of CX-5461, the First Direct and Selective Inhibitor of RNA Polymerase I, for Cancer Therapeutics. *ACS Medicinal Chemistry Letters*, **3**, 602-606.
